# Functionalized biomimetic nanoparticles are delivered from the nose to the brain for the synergistic targeted treatment of cerebral ischemia/reperfusion injury

**DOI:** 10.1093/rb/rbaf063

**Published:** 2025-06-27

**Authors:** Yuanyuan Wu, Huiyi Feng, Leying Gao, Xinyang Wang, Yue Hu, Xiaofang He, Qianqian Wu, Haolin Liu, Yu Long, Yuyu Fang, Nan Li

**Affiliations:** State Key Laboratory of Southwestern Chinese Medicine Resources, School of Pharmacy, Chengdu University of Traditional Chinese Medicine, Chengdu 611137, China; State Key Laboratory of Southwestern Chinese Medicine Resources, School of Pharmacy, Chengdu University of Traditional Chinese Medicine, Chengdu 611137, China; State Key Laboratory of Southwestern Chinese Medicine Resources, School of Pharmacy, Chengdu University of Traditional Chinese Medicine, Chengdu 611137, China; State Key Laboratory of Southwestern Chinese Medicine Resources, School of Pharmacy, Chengdu University of Traditional Chinese Medicine, Chengdu 611137, China; State Key Laboratory of Southwestern Chinese Medicine Resources, School of Pharmacy, Chengdu University of Traditional Chinese Medicine, Chengdu 611137, China; State Key Laboratory of Southwestern Chinese Medicine Resources, School of Pharmacy, Chengdu University of Traditional Chinese Medicine, Chengdu 611137, China; State Key Laboratory of Southwestern Chinese Medicine Resources, School of Pharmacy, Chengdu University of Traditional Chinese Medicine, Chengdu 611137, China; State Key Laboratory of Southwestern Chinese Medicine Resources, School of Pharmacy, Chengdu University of Traditional Chinese Medicine, Chengdu 611137, China; State Key Laboratory of Southwestern Chinese Medicine Resources, School of Pharmacy, Chengdu University of Traditional Chinese Medicine, Chengdu 611137, China; State Key Laboratory of Southwestern Chinese Medicine Resources, School of Pharmacy, Chengdu University of Traditional Chinese Medicine, Chengdu 611137, China; State Key Laboratory of Southwestern Chinese Medicine Resources, School of Pharmacy, Chengdu University of Traditional Chinese Medicine, Chengdu 611137, China

**Keywords:** cerebral ischemia/reperfusion injury, modified dextran, intranasal administration, microglia polarization

## Abstract

The pathology of cerebral ischemia/reperfusion (CIR) injury is complex. Additionally, single drugs have shown limited efficacy, and their delivery has encountered obstacles, such as the blood–brain barrier and poor targeting effects. Therefore, we designed a biomimetic nanoparticle: PR-M2/BED@BA was composed of boric acid ester-grafted dextran (BED) loaded with the drug baicalin (BA) and modified with an M2 microglial membrane and protamine sulfate (PR). Moreover, PR-M2/BED@BA homed to the brain lesion after entering the transnasal mucosa and released BA in response to the high content of reactive oxygen species in the microenvironment. *In vitro* studies have shown that BED has the ability to scavenge reactive oxygen species. PR-M2/BED@BA can rapidly release BA in an H_2_O_2_ environment, significantly enhancing the transport capacity across the nasal mucosal barrier and the uptake by microglia and neurons. *In vivo* studies showed that PR-M2/BED@BA significantly increased the amount of drug released into the brain, improved the neurobehavioral score and ameliorated pathological damage to the brain tissue in mice with global cerebral ischemia. This neuroprotective effect was related to the regulation of microglial polarization to reduce the inflammatory response, reduce microglial oxidative stress, and thus, reduce neuronal apoptosis. Overall, this study provides a new strategy for nasal–brain drug delivery and new ideas for the treatment of CIR injury and other neurological diseases.

## Graphical abstract

**Figure:**
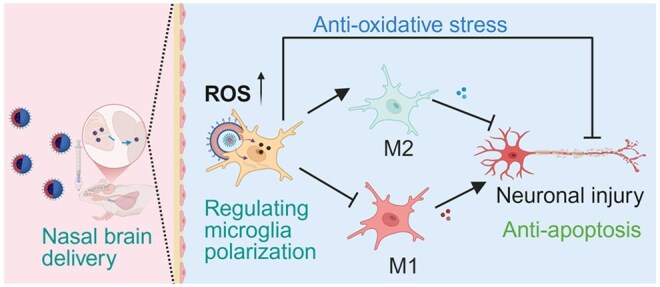

## Introduction

Ischemic stroke (IS) is the leading cause of death and disability worldwide and places great burdens on social and economic development [1]. At present, the main clinical treatments for IS are vascular recanalization therapies, such as intravenous thrombolysis and mechanical thrombectomy. However, blood flow recovery after ischemia may aggravate brain tissue damage and dysfunction, resulting in cerebral ischemia/reperfusion (CIR) injury [2, 3]. After IS, the microglia, as the resident immune cells in the brain, are the first cells to be activated and release inflammatory factors and chemokines, which direct peripheral immune cells to infiltrate the damaged blood–brain barrier and enter the focal area, further amplifying the inflammatory response [4]. These inflammatory mediators induce neuronal apoptosis through related pathways [5, 6]. Additionally, these inflammatory mediators also induce mitochondrial dysfunction, increase the release of reactive oxygen species (ROS) and promote oxidative stress. This excessive accumulation of ROS destroys the structures and functions of macromolecules such as proteins and lipids and stimulates caspase-dependent programmed cell death. Furthermore, the impaired cells release damage-associated molecular patterns, activate pattern recognition receptors on immune cells such as microglia and aggravate secondary inflammatory reactions [7]. Collectively, these events aggravate brain injury. Therefore, developing multitarget treatment methods to address the complex pathology of CIR injury is very important. Baicalin (BA) is a natural flavonoid with a wide range of pharmacological activities, including inhibiting oxidative stress, reducing inflammatory infiltration and exerting antiapoptotic effects [8]. However, free BA is poorly soluble and has low bioavailability, which limit its wide application. Therefore, new, efficient strategies to deliver BA must be developed.

Encapsulating drugs in biomimetic cell membrane using nanotechnology approaches has provided a broad strategy for drug delivery to the brain. The CX3C chemokine receptor1 (CX3CR1) receptor on the M2 microglial cell membrane responds to chemokines and regulates the migration of cells to inflammatory sites [9]. For therapeutic applications, M2 microglial membranes can neutralize proinflammatory factors and inhibit neuroinflammation [10]. In addition, administering drugs nasally for the treatment of encephalopathy has unique advantages, as this route of administration provides targeting effects, the ability to cross the blood–brain barrier and a rapid onset of action. Drugs can be delivered directly to the brain after nasal administration through the olfactory nerve and olfactory mucosal epithelial pathways to increase the drug concentration in the brain tissue [11]. However, the nasal mucosa is one barrier to the direct transport of drugs to the brain, as drugs must cross this layer to reach the epithelial surface and be absorbed; otherwise, the drug is cleared by the cilia [12]. Researchers have modified the surface of nanocarriers with cell-penetrating peptides that promote membrane penetration to overcome the nasal mucosal barrier. For example, protamine sulfate (PR) is an arginine (Arg)-rich cationic peptide that can increase the transmucosal permeability of drugs and has been used to deliver nucleic acids, proteins and NPs [13–15]. In addition to enhancing brain-targeted delivery, specific drug release remains a challenge. The macromolecular polysaccharide dextran has good biocompatibility and has been widely incorporated into drug carriers [16]. Chemically modified polysaccharides are environmentally friendly and can accelerate drug release in response to environmental stimuli, such as changes in pH, ROS levels or temperature [17–20]. Borate esters are commonly used as ROS-responsive functional groups. In a high-ROS environment, borate ester polymers can dissociate to accelerate drug release [21].

Inspired by these findings, we proposed a targeted, biomimetic nasal nanopreparation for the synergistic treatment of CIR injury via “one stone and three birds.” First, BA and the M2 microglial membrane exert anti-inflammatory effects by regulating microglial phenotypic polarization. Second, BA and the nanocarrier exert antioxidative effects by reducing the excess ROS levels. Third, the nanocarrier further reduces neuronal damage by regulating the microglia and oxidative stress. We first synthesized the ROS-responsive polymer boric acid ester-grafted dextran (BED), which self-assembled with poloxamer 188 to form an NP core loaded with the hydrophobic drug BA, to achieve this goal. Furthermore, the M2 microglial membrane was used to modify the NP surface upon coextrusion to form a core–shell bionic nanocarrier. Finally, through electrostatic adsorption, the positively charged PR wrapped the negatively charged biomimetic nanocarrier to obtain PR-M2/BED@BA. The nasal permeability of the nanocarrier increased after nasal administration due to the presence of PR, after which PR-M2/BED@BA crossed the nasal mucosal barrier and entered the brain. Then, the M2 microglial membrane mediated the homing of the nanocarrier to ischemic brain tissue, where it was specifically taken up by microglia. In this microenvironment, which contained high levels of ROS, PR-M2/BED@BA ultimately released BA.

## Materials and methods

### Materials

4-(Hydroxymethyl) phenylboronic acid pinacol ester (H830167), *N*, *N′*-carbonyldiimidazole (N805050), dextran (MW 10 000 g/mol, D992645), 4-dimethylaminopyridine (D807273), poloxamer 188 (P981753), coumarin 6 (C804226) and hydrogen peroxide (H792077) were purchased from Shanghai Macklin Biochemical Technology Co., Ltd. (Shanghai, China). Protamine sulfate (P4020) and dihydroethidium (DHE) (D7008-10) were purchased from Sigma-Aldrich Company (Shanghai, China). iNOS (ab283655), Bcl (ab182858), Bax (ab32503) and Caspase-3 (ab184787) antibodies and goat anti-rabbit IgG-HRP (ab205718) were purchased from Abcam Plc (Britain). The CD206 (24595) antibody was purchased from Cell Signaling Technology Company (America). Alexa Fluor ^®^ 488 donkey anti-rabbit IgG (A32790) was purchased from Life Technologies Company (America). The iba1 (OB-PGP049-01) antibody, Alexa Fluor ^®^ 594 goat anti-guinea pig IgG (OB-GP594-50) were purchased from Oasis Biotechnology Co., Ltd (Hangzhou, China). IL-4(E-EL-MOO43c), IL-10 (E-EL-M0046), TNF-α (E-EL-M3063), SOD (E-BC-K020-M), GSH (E-BC-K030-M) and an Annexin V-FITC/PI apoptosis kit (E-CK-A211) were purchased from Elabscience Biotechnology Co., Ltd. (Wuhan, China). The MDA Kit (A003-1-2) was purchased from Nanjing Jiancheng Bioengineering Institute (Nanjing, China). A BCA protein assay kit (P0012), a ROS detection kit (C1038), DiO (C1038) and DiD (C1039) were purchased from Shanghai Beyotime Biotechnology Co., Ltd. (Shanghai, China). Arg antibody (GB115724), IL-4 (GB300005), DAPI (G1407), CCK8 (G1613) and cell culture reagents were purchased from Wuhan Sevier Biotechnology Co., Ltd. (Wuhan, China). CX3CR1 antibody was purchased from Wuhan Sanying Biotechnology Co., Ltd.

### Synthesis and characterization of BED

The following ROS-responsive materials were initially synthesized [21]. First, 468.22 mg of 4-(hydroxymethyl) phenylboronic acid pinacol ester (PBAP) and 648.60 mg of N, N'-carbonyldiimidazole (CDI) were reacted in anhydrous dichloromethane for 1 h. The reaction mixture was then washed with water and a saline solution and activated PBAP-CDI was obtained after removal of the solvent via rotary evaporation. Additionally, 250 mg of PBAP-CDI, 45 mg of dextran and 100 mg of 4-dimethylaminopyridine (DMAP) were reacted in anhydrous dimethyl sulfoxide for 24 h. The product was dialyzed in a dialysis bag (MWCO 1000 Da, JielePu, USA) for 24 h, and then, freeze-dried to obtain BED as a white solid powder. BED was characterized via proton nuclear magnetic resonance spectroscopy (^1^H NMR; Bruker Avance Neo, 600 MHz; Bruker, Germany) and Fourier transform infrared (FTIR) spectroscopy (BUCHIN-500; Buchiglasuster, Switzerland).

### Preparation and characterization of PR-M2/BED@BA

One milligram of BA and 10 mg of BED were dissolved in 1 mL of a mixture of formamide and methanol (1:1, v/v), and this mixture was slowly added to 5 mL of poloxamer 188 (0.5%, w/v). Then, the mixture was dialyzed in a dialysis bag (MWCO 1000 Da) for 12 h to yield BED@BA. M2/BED@BA was subsequently prepared in two steps. (i) BV2 cells were induced with 40 ng/mL interleukin 4 (IL-4) for 24 hr [22] to generate M2-type BV2 cells. The cells were collected by centrifugation, suspended in TM buffer solution, lysed by ultrasonication and added to a 1 mol/L sucrose solution. After centrifugation (2000 × g, 10 min), the supernatant was collected and centrifuged again (20 000 × g, 30 min) and the precipitate from this step was collected the M2-BV2 cell membrane. Finally, the total protein content in the membrane fraction was determined using a BCA kit [23]. (ii) M2/BED@BA was obtained by mixing the M2-BV2 cell membrane with BED@BA with ultrasonication for 1 min, followed by 10 passages through a liposome extruder (61000, Avestin, Germany) [24]. Subsequently, 1 mg/mL PR was mixed with M2/BED@BA for an incubation overnight with stirring to obtain PR-M2/BED@BA [25].

The expression of the M2 signature proteins was examined via immunofluorescence staining to determine whether M2-BV2 cell induction was successful. Additionally, dynamic light scattering (DLS; Litesizer 500, Anton Paar, Austria) was used to measure the diameter and zeta potential of the NPs. The morphological appearance of the NPs was characterized by transmission electron microscopy (TEM) (Tecnai G2 12, Fel, USA). Furthermore, the M2-BV2 cell membranes were labeled with DiO and the core of the NPs was labeled with DiD, and their colocalization was observed via confocal laser scanning microscopy (CLSM; FV-OSR, OLYMPUS, Japan). The total protein content of the M2-BV2 cell membranes, M2/BED@BA and PR-M2/BED@BA was analyzed by SDS-PAGE. Western blotting (WB) was performed to analyze the retention of the specific proteins Arg, CD206 and CX3CR1 on the M2 microglial cell membranes in the NPs. BA, the M2-BV2 cell membranes, PR, BED@BA, M2/BED@BA, and PR-M2/BED@BA were also analyzed by FTIR. The stability of the preparation was investigated. PR-M2/BED@BA was placed in a refrigerator at 4°C, and the particle size and potential of each group of NPs were detected by DLS on days 1, 4, 8 and 12.

### ROS-responsive release of BA from PR-M2/BED@BA

BED@BA, M2/BED@BA and PR-M2/BED@BA were dissolved in 1 mL of phosphate-buffered saline (PBS), placed in a dialysis bag (MWCO 1000 Da) and dialyzed against 10 mL of PBS or 10 mL of PBS containing 1 mM hydrogen peroxide (H_2_O_2_). Samples of the external buffer were collected at 0.5, 1, 2, 4, 6 and 9 h, after which the external buffer was replenished with an equal amount of PBS, and the BA concentration in each of the samples was determined using a UV spectrophotometer. H_2_O_2_ (1 mM) was added to PR-M2/BED@BA, and the appearance of the preparation was observed by imaging at 0 min, 5 min, 15 min, 30 min, 1 h and 2 h, respectively. The change of diameter was detected by DLS. In addition, after the incubation with H_2_O_2_ for 2 h, the nanostructure of the preparation was observed by TEM.

### Cell culture and model establishment

BV2, PC12 and Calu-3 cells were purchased from Wuhan Sevier Biotechnology Co., Ltd, and cultured in an incubator at 37°C with 95% air and 5% CO_2_. For all the cells, the culture media consisted of 90% high-glucose DMEM (v/v), 10% FBS and a 1% penicillin and streptomycin solution.

An *in vitro* nasal mucosal model was established to explore the ability of the NPs to permeate the nasal mucosal barrier [26]. First, Calu-3 cells (1 × 10^5^) were seeded in the upper chamber of a transwell and cultured for 5 days, after which the culture was changed to a gas–liquid culture for 2 days. The transepithelial electrical resistance (TEER) was subsequently detected with a cell resistance meter (Millicell ERS-2, Millicell, USA). A dense cell monolayer formed when the TEER was greater than 500 Ω cm^2^, indicating successful establishment of the Calu-3 model. Free chlorin e6 (C6) and C6-labeled NPs (at a concentration equivalent to 5 μM C6) were added to the upper chamber of a transwell. After incubation for 4 h, the apparent permeability coefficient (*P*_app_) of the NPs in each group was calculated [27]. Then, a coculture model of Calu-3 and BV2 cells was established to further explore the uptake of NPs by BV2 cells after they crossed the nasal mucosa. Using the Calu-3 model, BV2 (1 × 10^5^) cells were inoculated in the lower chamber of a transwell and cultured overnight.

The oxygen–glucose deprivation/reoxygenation (OGD/R) model of BV2 cells was established as follows. First, BV2 cells were exposed to an oxygen–glucose deprivation environment (1% O_2_, 5% CO_2_ and 94% N_2_) in FBS- and sugar-free medium for 1 h. The sugar-free medium was discarded, complete medium was added and the cells were then placed in a normal cell incubator for 24 h of reoxygenation at 37°C [28]. NPs (200 μg/mL) were then added to the media of each group after reoxygenation and incubated with the BV2 cells for 24 h. The PC12 cell OGD/R model was established in the same manner.

A transwell coculture model of BV2 cells and PC12 cells was established [29]: BV2 cells (1 × 10^5^) were inoculated in a transwell chamber, and PC12 cells (1 × 10^6^) were inoculated in the lower chamber. After OGD/R, the cells were cocultured for 24 h. BV2 cells were incubated with NPs (200 μg/mL) for 24 h, washed with PBS, and then, seeded above PC12 cells.

### Cellular uptake

C6-labeled NPs were added to the upper chamber of a transwell and incubated for 4 h to establish the Calu-3 and BV2 cell coculture model. The fluorescence intensity of the BV2 cells was then detected with a high-content analysis system (Image Xpress Micro Confocal, Molecular Devices, USA).

BV2 cells and PC12 cells were subjected to OGD/R, and BED@C6, M2/BED@C6 or PR-M2/BED@C6 (at a concentration equivalent to 5 μM C6) was added to further explore the cell-targeting ability of the NPs *in vitro*. After the incubation with the BV2 and PC12 cells for 0.5, 1, 2 or 4 h, the cells were washed with PBS three times and fixed with 4% paraformaldehyde for 30 min, after which the intracellular fluorescence was detected by CLSM.

### Flow cytometry

After the BV2 and PC12 cells were incubated with the NPs for 4 h, flow cytometry (FCM) (FACSCanto II, BD Biosciences, USA) was used to quantify the mean intracellular fluorescence intensity. Then, the fluorescent probes Annexin V-FITC and PI were added to the PC12 cells induced with BV2 cell-conditioned medium, and PC12 cell apoptosis was detected by FCM according to the instructions provided with the kit.

### Intracellular ROS levels

The fluorescent probe DCFH-DA was added to NP-treated OGD/R model BV2 cells and incubated for 30 min, after which the intracellular ROS levels were visualized with a high-content analysis system.

### Animal modeling

As a method to study the neuroprotective effect of these NPs on transient global IS, KM mice were used to establish a mouse global cerebral ischemia model via bilateral common carotid artery ligation (BCAL) according to the method of Gao [30–32] with slight modifications. BED@BA, M2/BED@BA, PR-M2/BED@BA (at a concentration equivalent to 5 mg/kg BA) or edaravone (3 mg/kg) was administered via intranasal drip for three consecutive days, after which BCAL was performed [33, 34]. First, the bilateral common carotid arteries of the mice were exposed and clamped with arterial hemostatic forceps for 30 min to cause cerebral ischemia, after which the arterial hemostatic forceps were loosened for 10 min twice to allow reperfusion. A laser diffusion flow imaging system (RFLSI III, RFLSI, China) was used to monitor the changes in cerebral blood flow (CBF) in the designated regions of the mice before BCAL, 5 min after BCAL and after 5 min of reperfusion [35]. All animal experimental protocols were reviewed by the Experimental Animal Welfare Ethics Committee of Chengdu University of Traditional Chinese Medicine (approval number 2024199).

### Biological distribution

Next, small animal live imaging experiments were performed to assess the accumulation of NPs in the brains of the BCAL model mice after passing through the transnasal mucosa. The NPs were labeled with sulfo-cyanine 7 (Cy7), and BCAL model mice were intranasally instilled with BED@Cy7, M2/BED@Cy7, PR-M2/BED@Cy7 or free Cy7 (each at a concentration equivalent to 2 μM Cy7). At 1, 3, 6 and 9 h after administration, fluorescence imaging of the mice was performed using a multifunctional imaging system (UVP iBox Scientia, Analytik Jena, Germany), and the average fluorescence intensity in the brain of each mouse was quantified. The mice were sacrificed at 9 h, and the brain tissues were collected for *ex vivo* fluorescence imaging.

### Behavioral assays

Twenty four hours after BCAL modeling, the Zea-Longa scoring method was used to assess the degree of nerve damage to the mice in each of the NP groups; the higher the score was, the greater the degree of nerve function deficit [36]. Additionally, a balance beam experiment was used to assess the motor coordination and balance abilities of the mice in each group; again, the higher the score was, the greater the degree of balance function deficit [37]. Fourteen days after modeling, the mice were subjected to behavioral field experiments in the open field. The activities of the mice were recorded by behavioral trajectory software (XR-VT, Xinruan, China) for 10 min. The observation indices analyzed to assess the independent locomotor ability of the mice were the total distance traveled and the average movement speed.

### Hematoxylin–Eosin and Nissl staining

After the Zea-Longa scoring and the balance beam experiments, the mice were euthanized, and the brains were quickly removed, fixed with 4% paraformaldehyde, embedded in paraffin and sectioned for Hematoxylin–Eosin (HE) and Nissl staining.

### Western blotting

The brain tissues of the mice were homogenized and lysed, and the total protein content was measured using a BCA protein kit. After separation via SDS–PAGE, the proteins were transferred to a membrane, blocked with 5% bovine serum albumin for 2 h, washed and incubated with primary antibodies, including Arg, inducible nitric oxide synthetase (iNOS), ionized calcium binding adaptor molecule-1 (iba1), Caspase-3, B-cell lymphoma (Bcl), Bcl2-associated X protein (Bax) and β-actin antibodies, overnight at 4°C. After washing, the membranes were incubated with the secondary antibody. Antibody binding was finally detected with an enhanced chemiluminescence kit, and the gray value was analyzed with ImageJ software.

### Immunohistochemistry

Immunohistochemical staining of paraffin-embedded brain tissue was performed to detect the expression of Caspase-3, Bcl and Bax protein in the mouse cerebral cortex.

### Immunofluorescence staining

BV2 cells were fixed, and brain tissue sections were dewaxed and rehydrated for the immunofluorescence analysis. Then, the cells and tissue sections were permeabilized, blocked and incubated with primary antibodies against iba1, iNOS and Arg overnight at 4°C. After being washed, the cell and tissue samples were incubated with secondary antibodies against Alexa Fluor 488 and Alexa Fluor 594, and the nuclei were stained with DAPI. The expression of Arg, iNOS and iba1 in BV2 cells and brain tissues was visualized using a fluorescence microscope (BX53, Olympus, Japan).

### Enzyme-linked immunosorbent assays

The levels of the inflammatory factors tumor necrosis factor-α (TNF-α), IL-4 and interleukin 10 (IL-10) in brain tissue homogenates and BV2 cell supernatants were measured according to the instructions of the enzyme-linked immunosorbent assay (ELISA) kits. The expression levels of the oxidative stress indicators malondialdehyde (MDA), superoxide dismutase (SOD) and glutathione (GSH) in brain tissue homogenates and BV2 cell lysates were also detected using ELISA kits.

### TUNEL and dihydroethidium staining

TUNEL and DHE staining were performed to measure the levels of apoptosis and ROS in the brains of the BCAL mice after NPs treatment.

### Biosafety

We verified the safety of the NPs *in vivo*. Healthy mice were intranasally administered saline, BED@BA, M2/BED@BA or PR-M2/BED@BA (at concentration equivalent to 5 mg/kg BA). After 7 consecutive days of treatment, the heart, liver, spleen, lung, kidney and nasal mucosa of the mice were collected for HE staining. In addition, serum was collected to measure ALT (Alanine aminotransferase), ALB (Albumin) and AST (Aspartate aminotransferase) levels as indicators of liver function, and UA (Uric acid), UREA and CREA (Creatinine) levels as indicators of renal function; whole blood samples were collected for routine blood tests, with HCT (Hematocrit), HGB (Hemoglobin) and WBC (White blood cell count) as indicators.

### Statistical analysis

The data were analyzed by one-way ANOVA using GraphPad Prism version 10.0 software. All the data are presented as the means ± standard deviations (SDs), and *P* < 0.05 was considered to indicate statistical significance.

## Results and discussion

### Physicochemical characterization of the nanopreparation

The ^1^H NMR spectrum of BED was shown in Supplementary Figure S3. BED had 12 main peaks, of which a–h showed the main peaks of the dextran molecule, and i–l showed the main peak of the PABA-CDI molecule. FTIR spectroscopy revealed that BED and dextran had the same O–H bond absorption peak at 3410 cm^−1^ and a C–H bond absorption peak at 2430 cm^−1^. In addition, BED also had a characteristic C = O absorption peak at 1700 cm^−1^ and a characteristic C-O absorption peak at 1260 cm^−1^ (Supplementary Figure S4). The above results indicated that PABA-CDI was successfully linked to the glucan skeleton and that BED was successfully synthesized.

**Figure 1.**
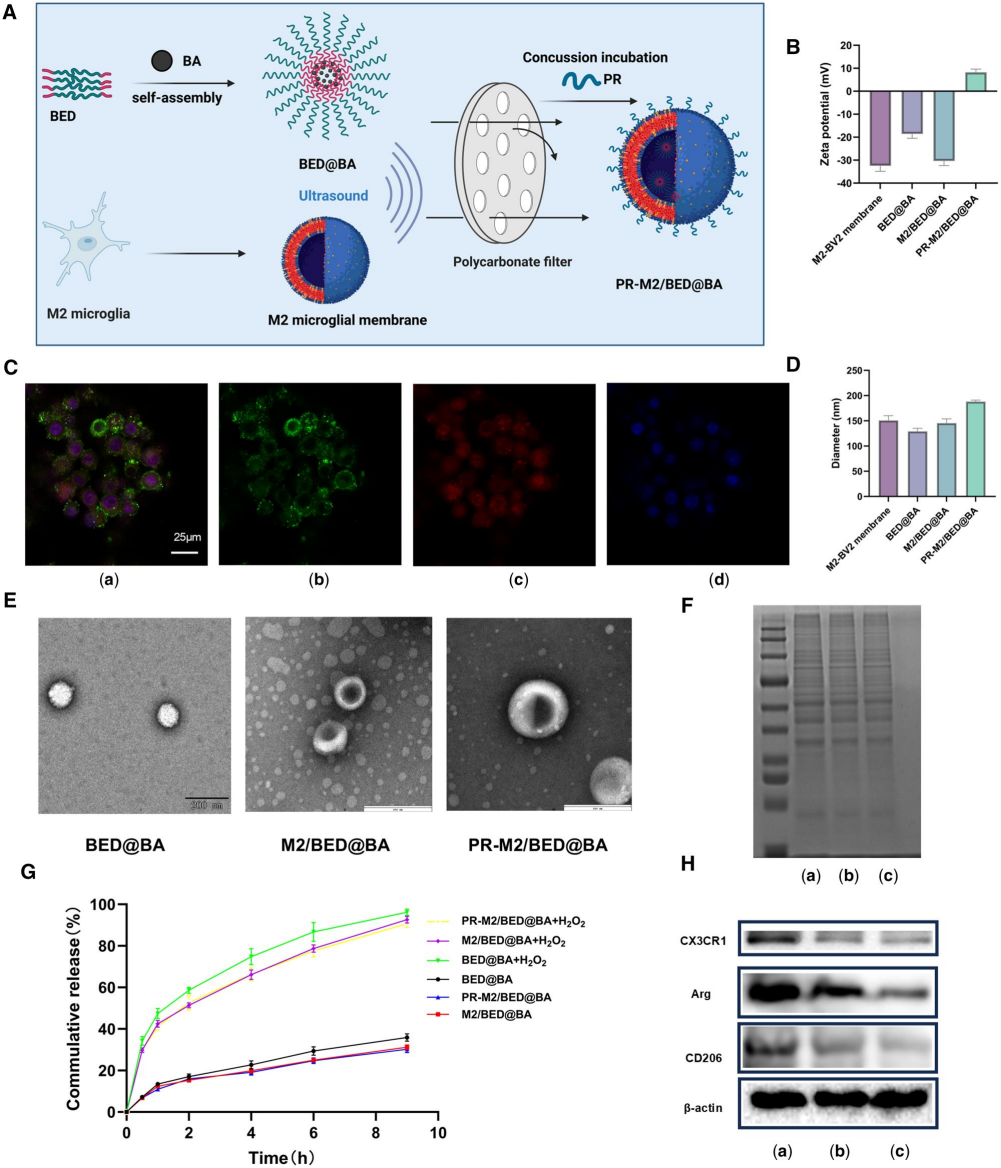
Preparation and characterization of PR-M2/BED@BA. (**A**) Preparation flow chart of PR-M2/BED@BA. (**B**) Zeta potential of NPs (*n* = 3). (**C**) Fluorescence colocalization image (a) Merged image (b) DiO-labeled M2-BV2 cell membranes (c) DiD-labeled nanoparticles (d) DAPI-labeled BV2 nucleus. (**D**) The diameters of NPs (*n* = 3). (**E**) TEM images of NPs. (**F**) SDS‐PAGE analysis of proteins. (a) M2 microglial cell membrane (b) M2/BED@BA (c) PR-M2/BED@BA (**G**) *In vitro* release curve of NPs (*n* = 3). (**H**) WB analysis of CX3CR1, Arg, CD206 and β-actin (*n* = 3). (a) M2 microglial cell membranes (b) M2/BED@BA (c) PR-M2/BED@BA.

The diameters (Figure 1D) and zeta potentials (Figure 1B) of the NPs were measured by DLS. The average diameter of BED@BA was 129 ± 5.06 nm, and the average zeta potential was −18.7 ± 1.55 mV. After being coated with the negatively charged M2-BV2 cell membranes, the average diameter of M2/BED@BA increased slightly to 145.2 ± 7.01 nm, and the average zeta potential decreased to −30.4 ± 1.64 mV. Furthermore, upon encapsulation by positively charged PR, the average diameter of PR-M2/BED@BA increased slightly to 186.4 ± 0.70 nm, and the zeta average potential increased to +8.23 ± 1.13 mV. After PR-M2/BED@BA was heated at 4°C for 12 days, the diameter increased slightly, but the zeta potential did not change significantly. These findings indicate that PR-M2/BED@BA can be stored stably for at least 12 days at 4°C (Supplementary Figure S10). TEM (Figure 1E) revealed that M2/BED@BA and PR-M2/BED@BA presented core–shell structures and larger NP sizes. The increase in the NP size and decrease in the NP surface potential indicated that the M2-BV2 cell membranes were successfully modified on the surface of the NPs, whereas the subsequent increase in the surface potential indicated that PR had been adsorbed onto the NP surface. CLSM allowed the visualization of green and red fluorescence that formed an encapsulated core–shell structure (Figure 1C), confirming that the BED@BA core was coated with the M2-BV2 cell membranes. The FTIR of spectrum BA (Supplementary Figure S5) revealed an O-H absorption peak at 3169.2 cm^−1^ and a C = O absorption peak at 1650.7 cm^−1^; however, these two characteristic peaks were almost undetectable in the BED@BA spectrum, indicating that BA was encapsulated in the core of the NPs. Upon modification with the M2 microglial membrane, the M2/BED@BA absorption peaks at 3359.2 cm^−1^ and 1639.9 cm^−1^ became more intense. Compared with those of M2/BED@BA, the PR-M2/BED@BA absorption peaks at 3316.9 cm^−1^ and 2898 cm^−1^ were weaker, whereas the peak at 1658.7 cm^−1^ was more intense. Importantly, PR has an O-H and N-H absorption peak at 3261.5 cm^−1^, a C-H absorption peak at 2900.2 cm^−1^ and a C = O absorption peak at 1650.7 cm^−1^.

Immunofluorescence staining revealed that the expression of the M2 microglia-specific proteins Arg and CD206 increased after 24 h of treatment with IL-4 (Supplementary Figure S1), indicating successful induction. The SDS–PAGE results (Figure 1F) showed that PR-M2/BED@BA retained the total proteins on the M2 microglial cell membranes. WB results (Figure 1H) showed that M2 microglia marker proteins (Arg and CD206) and the chemokine receptor CX3CR1 were expressed in PR-M2/BED@BA, indicating that PR-M2/BED@BA retained the specific proteins of the M2 microglial cell membranes.

The release of the ROS-responsive drug from the NPs was further evaluated. As shown in Figure 1G, after an incubation with H_2_O_2_ for 4 h, 74% of the BA was released from BED@BA, whereas only 22% of the BA was released in PBS, indicating that H_2_O_2_ accelerated the release of BA from the NPs. However, after an incubation with H_2_O_2_ for 4 h, the BA release rates of both M2/BED@BA and PR-M2/BED@BA were only 64%, indicating that the cell membrane and PR modifications hindered drug release from the NPs. As shown in Supplementary Figure S7, after the incubation with H_2_O_2_, PR-M2/BED@BA gradually changed from light blue opalescence to transparency. The DLS detection showed that the diameter of PR-M2/BED@BA became larger after the incubation with H_2_O_2_, and there were multiple small peaks (Supplementary Figure S8 and Supplementary Table S2). TEM showed that PR-M2/BED@BA was irregularly fragmented after incubation with H_2_O_2_ for 2 h (Supplementary Figure S9). These results indicate that PR-M2/BED@BA can respond to environmental H_2_O_2_ to rapidly release BA.

### Ability of PR-M2/BED@BA to cross the nasal mucosa and accumulate in the brain

We evaluated the toxicity of PR-M2/BED@BA to Calu-3, BV2 and PC12 cells to ensure the biosafety of the NPs *in vitro*. The results showed that after treatment with 200 μg/mL PR-M2/BED@BA, all three cell lines had survival rates greater than 80%, indicating that the nanocarrier had low cytotoxicity (Supplementary Figure S6).

**Figure 2.**
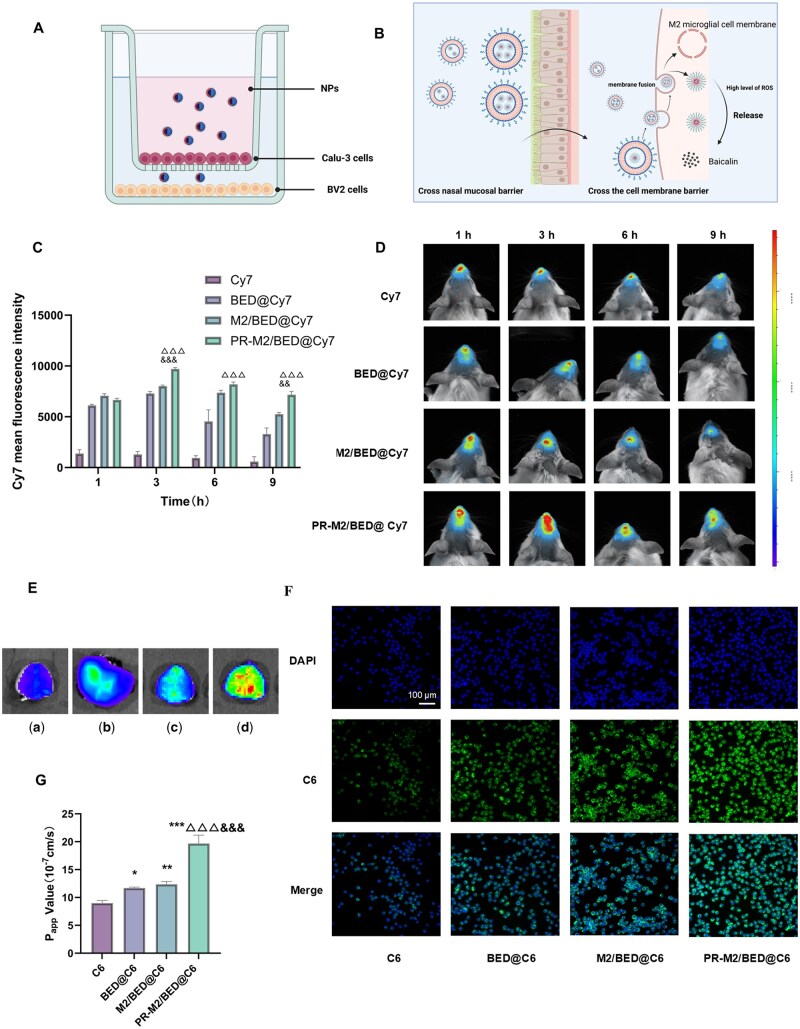
PR-M2/BED@BA was transnasally transported and enriched in the brain. (**A**) A schematic of the transwell model of BV2 and Calu-3 cells. (**B**) The schematic diagram of PR-M2/BED@BA from nose to brain. (**C**) The mean fluorescence intensity of Cy7 in the brains of the BCAL model mice was quantified as described in (**D**). (**D**) A multifunctional imaging system was used to detect the fluorescence intensity of Cy7 in the brains of the BCAL model mice (*n* = 3). ^△△△^*P* < 0.001 vs. BED@Cy7 group; ^&&&^*P* < 0.001 and ^&&^*P* < 0.01 vs. M2/BED@Cy7 group. (**E**) The distribution of Cy7 in the brain at 9 h after the nasal administration in the different NP groups. (a) Cy7, (b) BED@BA, (c) M2/BED@BA and (d) PR-M2/BED@BA. (**F**) Transmembrane uptake by BV2 cells in different NP groups in the transwell model. (*n* = 4). (**G**) *P*_app_ coefficients of the different NP groups in the transwell model. (*n* = 4) ***P < 0.001, **P < 0.01 and *P < 0.05 vs. C6 group; ^△△△^*P* < 0.001 vs. BED@C6 group; ^&&&^*P* < 0.001 vs. M2/BED@C6 group.

We further used a transwell culture model to explore the process by which NPs pass through the nasal mucosal barrier and are absorbed by BV2 cells (Figure 2A). Compared with that of the free C6 solution, the epithelial transport efficiency of the NPs in each group was significantly higher. Among them, the *P*_app_ of PR-M2/BED@C6 was the highest, at 1.963 × 10^−6 ^cm/s, which was significantly higher than those of the free C6 and other NP groups (Figure 2G). These findings suggest that the PR modification helps nanopreparation cross the nasal mucosal barrier. In addition, the uptake of NPs by BV2 cells after crossing the nasal mucosal barrier was assessed by high-content analysis system (Figure 2F). We found that, compared with BED@C6, M2/BED@C6 modified with the M2 cell membrane was better taken up by microglia, which may be related to the adhesion interactions among homologous cell surface proteins. After the PR modification, the uptake of PR-M2/BED@C6 by microglia increased further. These results indicate that the PR-M2/BED@C6 nanocarrier can effectively cross the nasal mucosal and cell membrane barriers with the aid of PR and the M2 microglial membrane, respectively.

After the intranasal administration of free Cy7 and Cy7-labeled NPs, the distribution of the NPs in the brains of the BCAL model mice was detected by a multifunctional imaging system at different time points. As shown in Figure 2D, the fluorescence was concentrated mainly in the nasal cavity of the mice in the free Cy7-treated group, and weak fluorescence was detected in the brain. In contrast, all the NP-treated mice presented stronger fluorescence in the brain, and the fluorescence signal in the PR-M2/BED@Cy7 treatment group was the strongest. In addition, the accumulation of fluorescent NPs in the brain was time dependent. The fluorescence intensity of PR-M2/BED@Cy7 was the highest at 3 h after administration, and the fluorescence intensity in the brain in this group was at least 1-fold higher than that in the BED@Cy7 group at 9 h. Therefore, the fluorescence intensity in the brain tissue was detected *in vivo* 9 h after nasal administration in subsequent experiments. These results were consistent with the *in vivo* data (Figure 2E), indicating that the fluorescence signal intensity and abundance of PR-M2/BED@Cy7 in brain tissue may have increased due to the M2 microglial membrane and PR modifications.

### Cellular uptake

**Figure 3.**
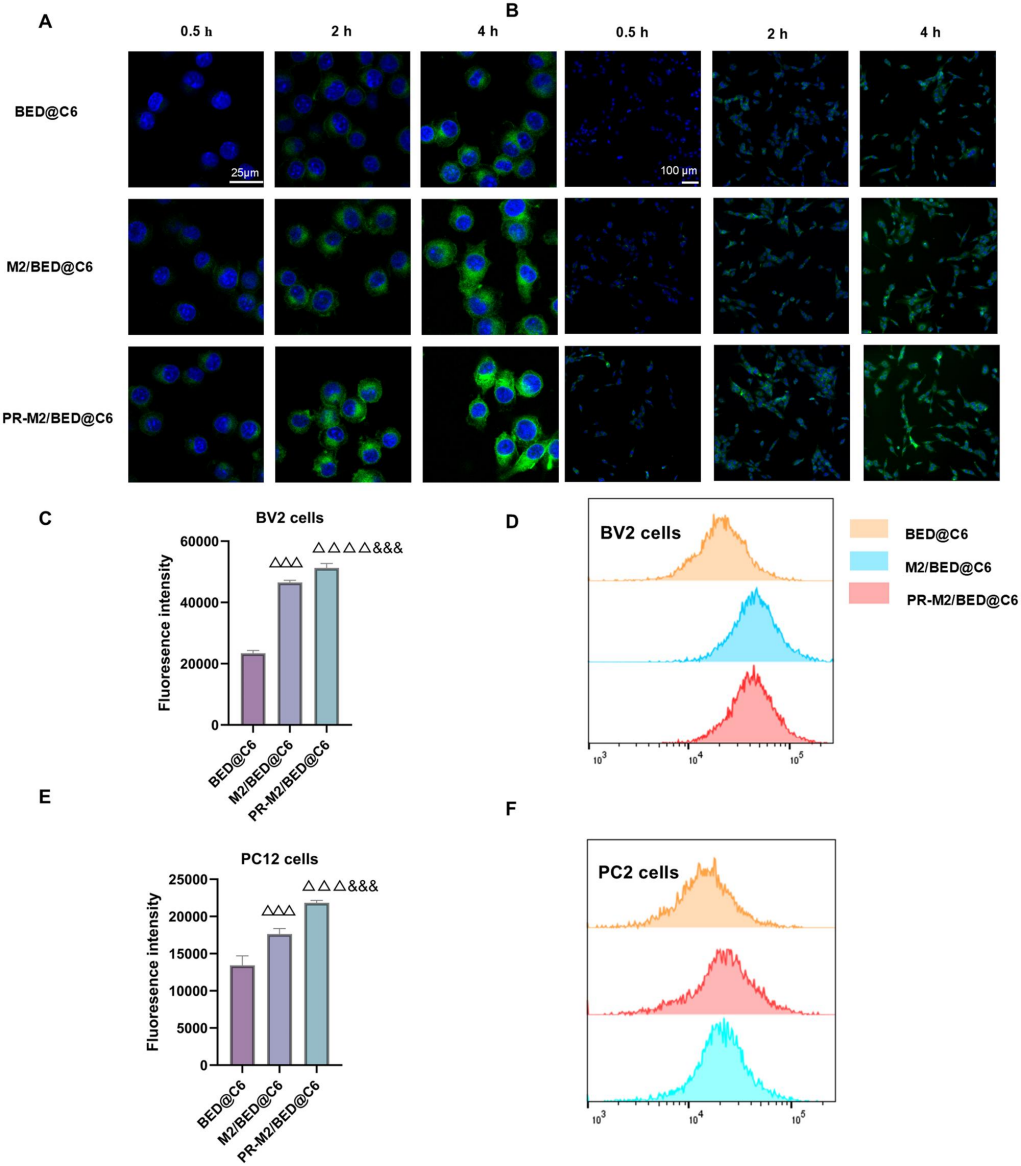
Cellular uptake of PR-M2/BED@BA. (**A**) CLSM was used to detect the uptake of NPs by OGD/R-treated BV-2 cells (*n* = 4). (**B**) CLSM was used to detect the uptake of NPs by OGD/R-treated PC12 cells (*n* = 4). (**C**) FlowJo v10.8.1 software was used to quantify the average fluorescence intensity of the data shown in (**D**) (*n* = 4). (D) FCM results for NP uptake by OGD/R-treated BV2 cells after an incubation for 4 h. (**E**) FlowJo v10.8.1 software was used to quantify the average fluorescence intensity of the data shown in (**F**) (*n* = 4). (**F**) FCM results for NP uptake by OGD/R-treated PC12 cells after an incubation for 4 h. ^△△△^*P* < 0.001 and ^△△△△^*P* < 0.0001 vs. BED@C6 group; ^&&&^*P* < 0.001 vs. M2/BED@C6 group.

First, the ability of the NPs to target OGD/R injury was evaluated *in vitro*. The NPs were labeled with C6, and NP uptake by BV2 and PC12 cells was investigated by CLSM and FCM. The results showed that NP uptake by BV2 and PC12 cells was time dependent. PR-M2/BED@C6 showed the highest uptake, and the rate of internalization of M2/BED@C6 was significantly higher than that of BED@C6 (Figure 3A and B). These data are consistent with the FCM results (Figure 3D and F). Notably, M2/BED@C6 was internalized at a greater rate by BV2 cells than by PC12 cells. This process may be mediated by adhesion interactions between specific cell surface proteins, which enhance the binding of the membrane-camouflaged NPs to cells of the same type. Furthermore, PR-M2/BED@C6 presented the highest rate of cellular internalization. The positively charged PR coating may cause the entire NP to become positively charged so that the NPs can participate in nonspecific electrostatic interactions with the negatively charged proteoglycans on the cell surface, which increases the NP uptake rate. These results confirm that encapsulation with the M2 microglial membranes increases the affinity of the NPs for these cells. These findings indicate that PR-M2/BED@C6 has the potential to target cells in ischemic tissues, especially microglia.

### Effects against is *in vivo*

**Figure 4.**
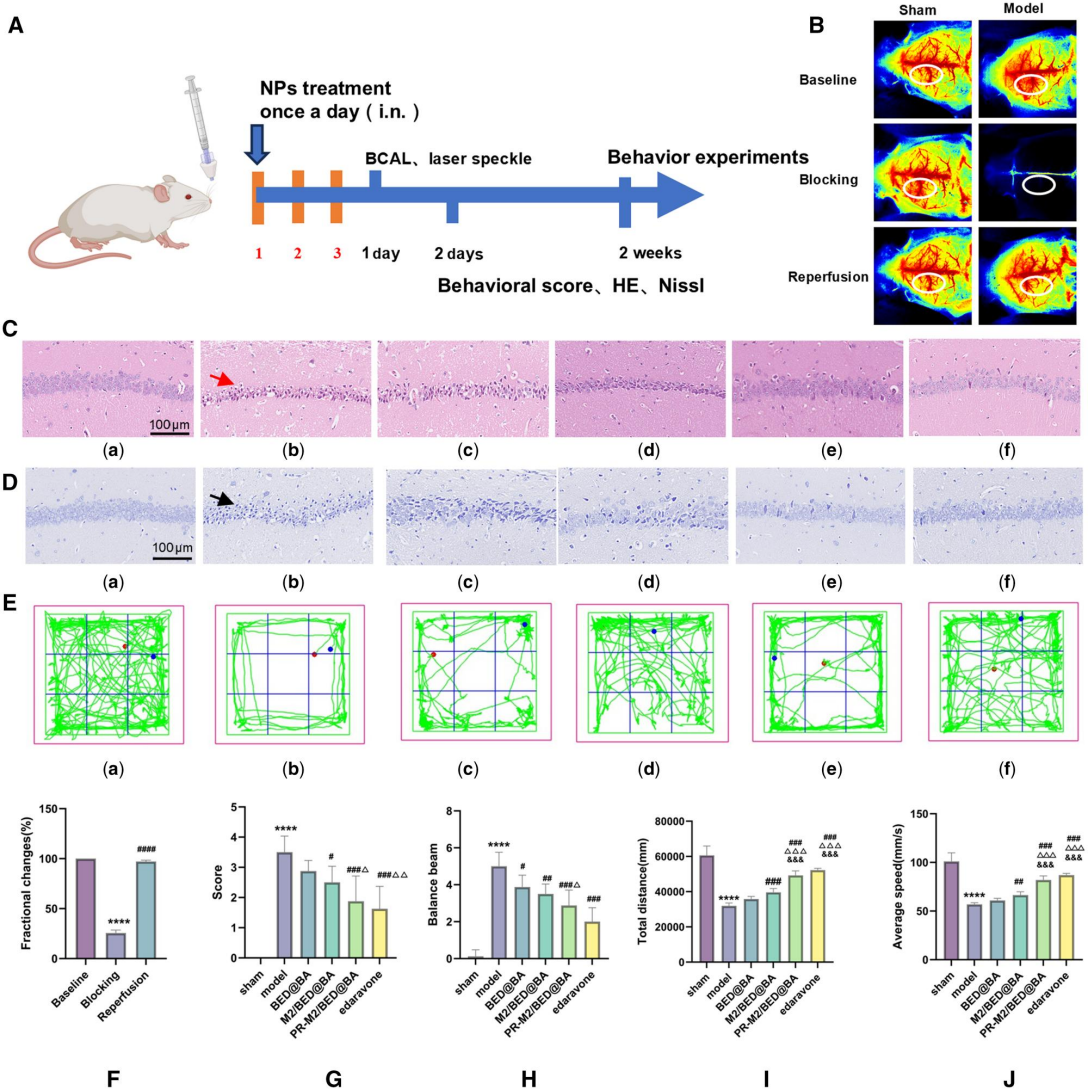
Therapeutic effect of PR-M2/BED@BA on BCAL mice *in vivo*. (**A**) Diagram of the treatment plan. (**B**) CBF changes observed under laser speckle blood flow imaging. Grouping (*n* = 4). (**C**) HE staining (*n* = 4). (**D**) Nissl staining (*n* = 4). (**E**) Open field experiment (*n* = 8). (a) Sham, (b) model, (c) BED@BA, (d) M2/BED@BA, (e) PR-M2/BED@BA and (f) edaravone groups. (**F**) Laser speckle blood flow statistics (*n* = 4). (**G**) The average neurological score of the BCAL mice after NP or edaravone treatment (*n* = 8). (**H**) Balance beam scores of the BCAL mice after NP and edaravone treatment (*n* = 8). The total distance traveled (**I**) and average speed (**J**) of the BCAL mice treated with NPs and edaravone in the open field test (*n* = 8). ****P < 0.0001 vs. sham group; ^####^*P* < 0.0001, ^###^*P* < 0.001, ^####^*P* < 0.01 and ^#^*P* < 0.05 vs. model group; ^△△△^*P* < 0.001, ^△△^*P* < 0.01 and ^△^*P* < 0.05 vs. BED@BA group; ^&&&^*P* < 0.001 vs. M2/BED@BA group.

As shown in Figure 4F, the CBF decreased significantly by >70% after occlusion compared with that before occlusion. After loosening the hemostatic forceps, the CBF increased and was not significantly different from that before ligation, indicating that the BCAL model was successfully established. Twenty-four hours after modeling, the Zea Longa score and balance beam test were used to evaluate the neurological impairment and motor coordination abilities of the mice. Compared with mice in the sham group, the Zea Longa and balance beam test scores of mice in the model group were significantly higher (Figure 4G and H). Compared with mice in the model group, the Zea Longa and balance beam test scores of the mice in the PR-M2/BED@BA group were significantly lower, and these mice showed neurological function recovery similar to that after treatment with the model drug edaravone. Immunohistochemical staining of the brain tissue sections was subsequently performed. After HE staining (Figure 4C), compared with those in the blank group, the pyramidal cells in the hippocampi of the model group had degenerated, the nuclei had shrunk, the number of basophils had increased, and the volume had decreased. The Nissl staining results revealed that the number of Nissl bodies in the hippocampi of the model group decreased (Figure 4D). Compared with the model group, each of the NPs and the positive control drug edaravone reduced brain tissue damage to varying degrees, and the improvements were the most obvious in the PR-M2/BED@BA and edaravone groups.

An open field experiment was performed 14 days after model induction to further explore how the NPs affect the autonomous movement abilities of mice after CIR injury. These results are shown in Figure 4E. Compared with the sham group, the total distance traveled and average speed of the model group were significantly lower, indicating that the BCAL model mice exhibited motor dysfunction (Figure 4I and J). Compared with the model group, the total distances traveled and average speeds of the M2/BED@BA, PR-M2/BED@BA and edaravone groups increased significantly to varying degrees. Additionally, no significant differences were observed between the PR-M2/BED@BA and edaravone groups. In summary, PR-M2/BED@BA has neuroprotective effects and has great potential for treating CIR injury.

### Regulation of microglial polarization

The microglial phenotype plays an important role in the neuroimmune system-mediated inflammatory response. Studies have shown that activated M1 microglia produce proinflammatory factors, such as TNF-α, matrix metalloproteinase 9 (MMP-9) and ROS, which aggravate neuronal damage. In contrast, M2 microglia produce anti-inflammatory factors, such as IL-4, which inhibit the inflammatory response and promote tissue recovery [38–40].

**Figure 5.**
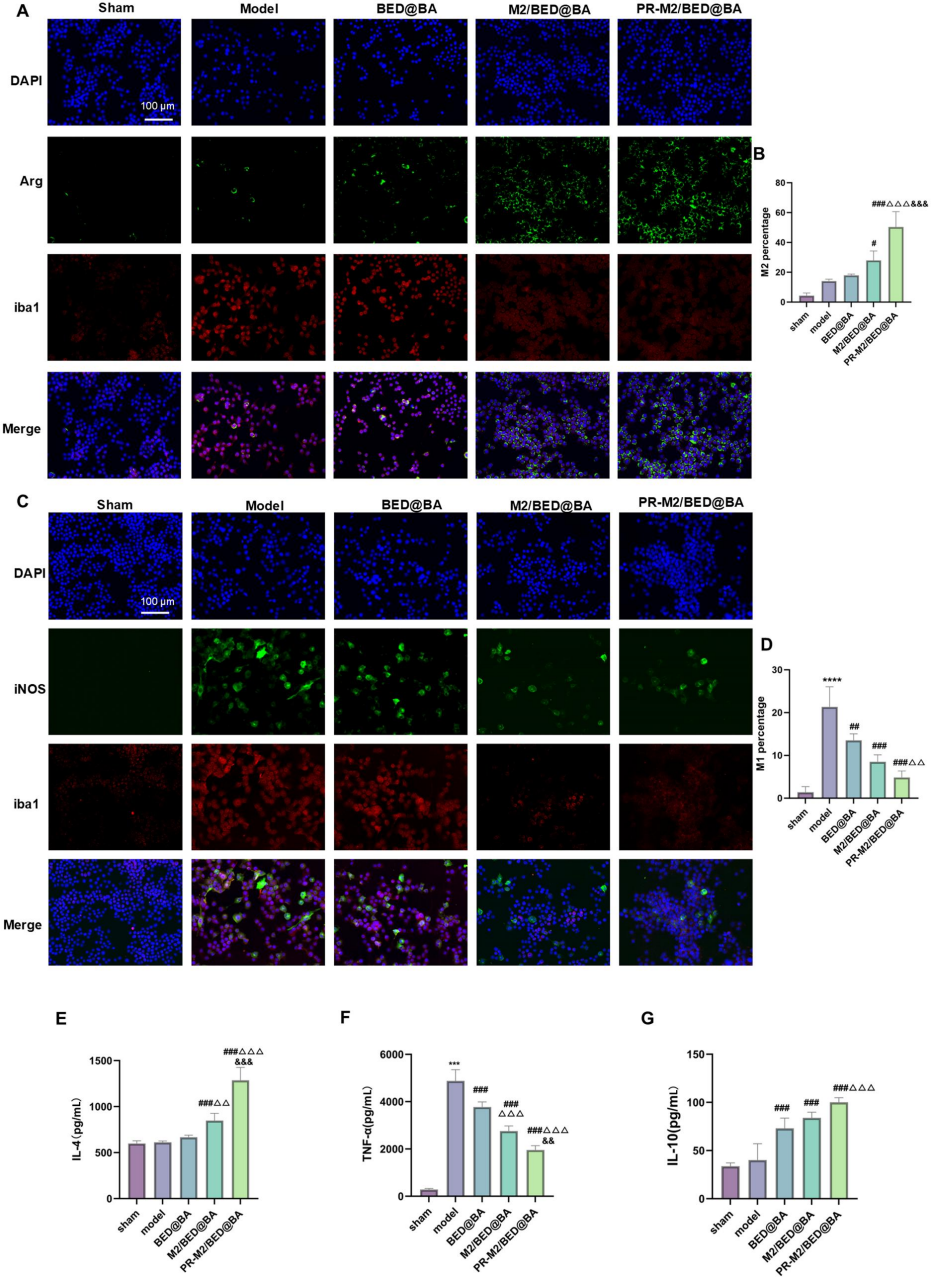
Microglial polarization and inflammatory factor release *in vitro* after NP treatment. (**A**) Immunofluorescence detection of the expression of the M2 signature protein Arg in BV2 cells subjected to OGD/R (*n* = 4). (**B**) ImageJ software was used to quantify the percentage of cells expressing Arg/iba1 (*n* = 4). (**C**) Immunofluorescence detection of the expression of the M1 signature protein iNOS in BV2 cells subjected to OGD/R (*n* = 4). (**D**) ImageJ software was used to quantify the ratio of iNOS/iba1 in the cells (*n* = 4). levels of the anti-inflammatory cytokines IL-4 (**E**) and IL-10 (**G**) in the supernatants of BV2 cells from different groups subjected to OGD/R and the relative expression levels of the proinflammatory cytokine TNF-α (**F**) (*n* = 6). ****P < 0.0001 and ***P < 0.001 vs. sham group; ^###^*P* < 0.001, ^##^*P* < 0.01 and ^#^*P* < 0.05 vs. model group; ^△△△^*P* < 0.001 and ^△△^*P* < 0.01 vs. BED@BA group; ^&&&^*P* < 0.001 and ^&&^*P* < 0.01 vs. M2/BED@BA group.

Therefore, we evaluated whether the NPs affected the polarization of microglia after CIR injury. In the *in vitro* BV2 cell model of OGD/R injury, the expression of M1 (iNOS/iba1) and M2 (Arg/iba1) marker proteins in each group was detected by immunofluorescence staining. Compared with the model group, the BED@BA, M2/BED@BA and PR-M2/BED@BA groups exhibited a reversal of the M1-type BV2 cell polarization caused by OGD/R to varying degrees and increased M2-type BV2 cell polarization (Figure 5A and C). Next, we studied the changes in the levels of proinflammatory and anti-inflammatory factors in the BV2 cell supernatant after treatment with different NPs by ELISA (Figure 5E–G). Compared with the model group, PR-M2/BED@BA significantly reduced the content of TNF-α in the BV2 cell supernatant and significantly increased the contents of IL-4 and IL-10. These data indicate that PR-M2/BED@BA can inhibit the release of inflammatory factors by M1-type BV2 cells and promote the release of anti-inflammatory factors by M2-type BV2 cells.

**Figure 6.**
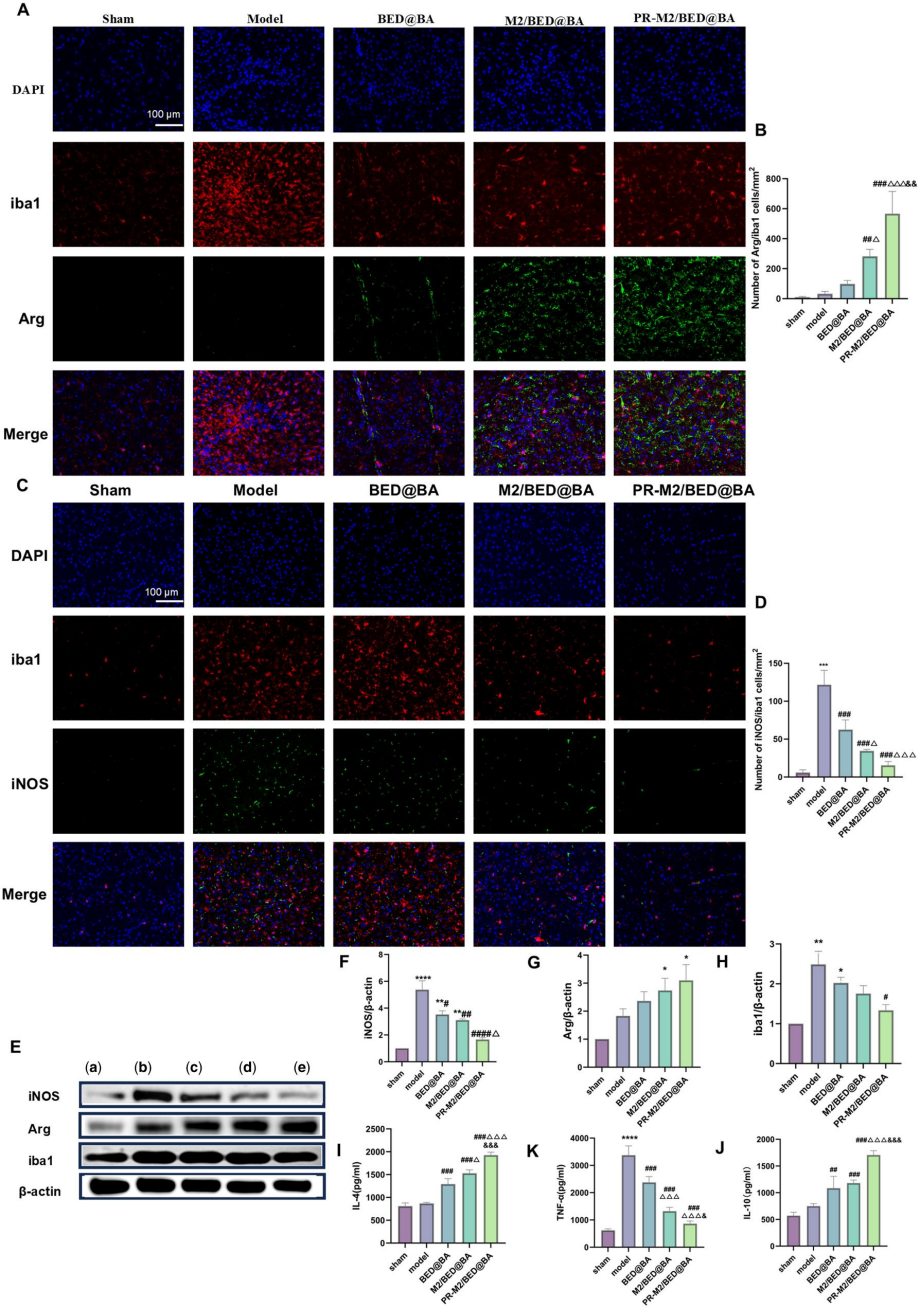
Microglial polarization and inflammatory factor release *in vivo* after NPs treatment. (**A**) Immunofluorescence detection of the expression of the M2 signature protein Arg in brain tissue (*n* = 4). (**B**) ImageJ software was used to quantify the number of cells expressing Arg/iba1 (*n* = 4). (**C**) Immunofluorescence detection of the expression of the M1 signature protein iNOS in brain tissue (*n* = 4). (**D**) ImageJ software was used to quantify the number of iNOS/iba1-producing cells (*n* = 4). (**E**) WB detection of iNOS, Arg and iba1 protein expression levels in brain tissues. ImageJ software was used to semiquantitatively analyze the grayscale values of iNOS (**F**), Arg (**G**) and iba1 (**H**) (*n* = 3). Groups: (a) sham, (b) model, (c) BED@BA, (d) M2/BED@BA and (e) PR-M2/BED@BA. Relative expression levels of the anti-inflammatory cytokines IL-4 (**I**) and IL-10 (**J**) and the proinflammatory cytokine TNF-α (**K**) in brain tissues from different NPs-treated groups. (*n* = 6) ****P < 0.0001, ***P < 0.001, **P < 0.01 and *P < 0.05 vs. sham group; ^####^*P* < 0.0001, ^###^*P* < 0.001, ^##^*P* < 0.01 and ^#^*P* < 0.05 vs. model group; ^△△△^*P* < 0.001 and ^△^*P* < 0.05 vs. BED@BA group; ^&&^*P* < 0.01 and ^&^*P* < 0.05 vs. M2/BED@BA group.

Encouraged by the results of the *in vitro* experiments, we subsequently performed *in vivo* experiments to verify that the NPs can regulate BV2 cell polarization. As shown in Figure 6A and C, compared with the numbers in the sham operation group, the numbers of M1-type (iNOS/iba1) and M2-type (Arg/iba1) microglia in the model group were greater, indicating that after IS, microglial polarization occurred, with M1-type polarization being predominant. After the intervention with the NPs, the number of M2-type (Arg/iba1) microglia increased, whereas the number of M1-type (iNOS/iba1) microglia decreased. Among them, PR-M2/BED@BA was the most efficacious treatment (Figure 6B and D). This finding was consistent with the WB results (Figure 6E). The PR-M2/BED@BA group presented significantly increased expression of Arg and significantly decreased expression of iNOS and iba1 (Figure 6F–H). Compared with the levels in the model group, the levels of the anti-inflammatory cytokines IL-10 and IL-4 in the PR-M2/BED@BA group were significantly increased, as determined by ELISA, and the level of the proinflammatory factor TNF-α was significantly decreased (Figure 6I and J). These data indicate that PR-M2/BED@BA can inhibit M1 microglial polarization, reduce the release of proinflammatory factors, promote the M2 polarization of BV2 cells, and increase the release of anti-inflammatory factors, thereby reducing the inflammatory response.

In general, the PR-M2/BED@BA nanocarrier can regulate the polarization of microglia to the M2 phenotype *in vitro* and *in vivo* and plays an anti-inflammatory role. The good therapeutic effects of PR-M2/BED@BA are partly due to the modification with the transmembrane peptide PR and the biomimetic cell membranes, which allow the nanopreparation to cross the nasal mucosal and cell membrane barriers and exhibit targeted enrichment. These phenomena are also closely related to the function of the M2 microglial membranes. The physiological characteristics of living cells are mostly mediated by ligand receptor proteins on the cell membrane. In CIR pathology, M2 microglia play a neuroprotective role by secreting biological signaling molecules such as anti-inflammatory factors to reduce the inflammatory response. The M2 microglial membranes retain these ligand receptor proteins, and thus, inherit their specific functions to regulate microglial polarization and inflammation to a certain extent.

### Reduction in oxidative stress

Oxidative stress caused by CIR injury is an important mechanism that leads to neuronal damage. High levels of ROS can cause inflammation and neuronal apoptosis, thereby aggravating brain damage [41]. BA has been reported to regulate succinate dehydrogenase (SDH)-mediated ROS production in early IS, resulting in antioxidative stress activity [42]. Previous experiments have shown that BED has good ROS-responsive drug release ability *in vitro*. Notably, *in vitro* cell experiments revealed that BED reversed the increase in ROS levels in BV2 cells caused by OGD/R to a certain extent. Compared with BED or BA alone, BED@BA was better able to scavenge ROS (Supplementary Figure S11). These findings indicate that BED and BA play synergistic roles in scavenging ROS.

**Figure 7.**
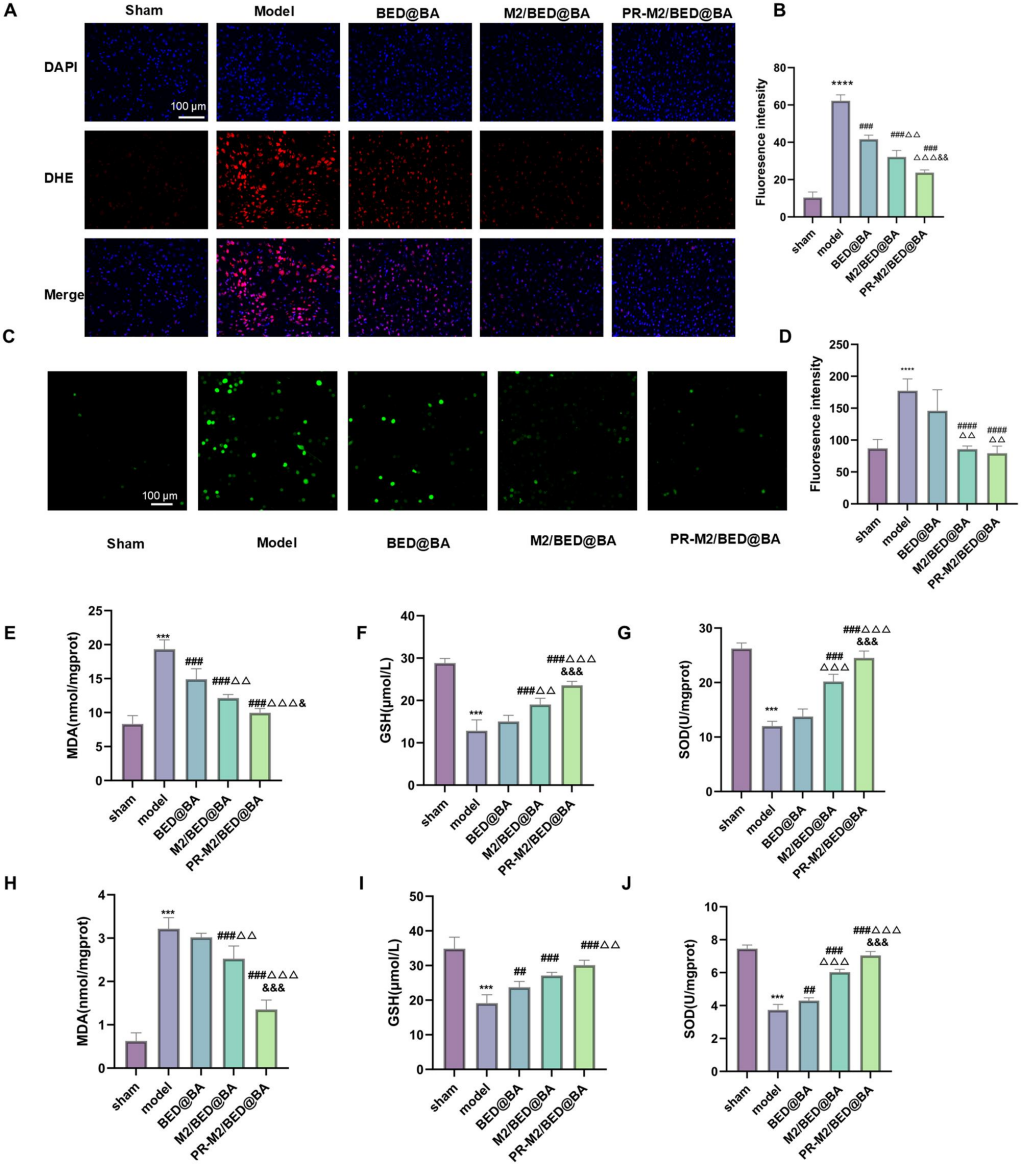
Antioxidative stress capacity of PR-M2/BED@BA. (**A**) DHE immunofluorescence staining of BCAL mice treated with different NPs (*n* = 4). (**B**) ImageJ software analysis of the data in (A) Quantification of the red fluorescence intensity (*n* = 4). (**C**) Fluorescent probes were used to detect ROS levels in BV2 cells subjected to OGD/R after treatment with different NPs. (**D**) ImageJ software analysis of the data in (**C**) Quantification of the green fluorescence intensity (*n* = 4). relative expression levels of the oxidative stress indicators MDA (**E**), GSH (**F**) and SOD (**G**) in the brain tissues of BCAL mice and MDA (**H**), GSH (**I)** and SOD (**J**) levels in BV2 cells subjected to OGD/R after treatment with different NPs groups (*n* = 6). ****P < 0.0001 and ***P < 0.001 vs. sham group; ^####^*P* < 0.0001, ^###^*P* < 0.001 and ^##^*P* < 0.01 vs. model group; ^ΔΔΔ^*P* < 0.001 and ^ΔΔ^*P* < 0.01 vs. BED@BA group; ^&&&^*P* < 0.001, ^&&^*P* < 0.01 and ^&^*P* < 0.05 vs. M2/BED@BA group.

Therefore, we further explored the antioxidative stress effect of the NPs *in vitro* and *in vivo* in a mouse BCAL model. Confocal imaging revealed that the green fluorescence intensity in the OGD/R model BV2 cells was significantly increased. After quantification, the green fluorescence decreased to varying degrees after NP treatment, and PR-M2/BED@BA showed the greatest ability to scavenge ROS (Figure 7C). The levels of the oxidative stress markers MDA, SOD and GSH in BV2 cells were detected via ELISA (Figure 7H–J). Compared with those in the sham group, the contents of the antioxidants SOD and GSH in the model BV2 cells were significantly lower, and the content of the lipid peroxide marker MDA was significantly higher. These changes were reversed in the PR-M2/BED@BA group. Moreover, we used the probe DHE to measure the level of ROS in the cortical regions of the mice (Figure 7A). Consistent with the *in vitro* results, the model group presented the strongest red fluorescence, and the PR-M2/BED@BA group presented the most significant inhibition of ROS production. PR-M2/BED@BA significantly reduced the content of MDA in the brain tissue (Figure 7E) and significantly increased the contents of SOD and GSH (Figure 7F and G). These results confirmed that the PR-M2/BED@BA nanodrug delivery system can effectively increase the ability of BA to scavenge ROS and counteract oxidative stress. Two main causes of this synergistic effect were identified. First, owing to the M2-BV2 cell membrane coating, the rate of NP internalization by BV2 cells was greatly improved. Conversely, these lesion microenvironments contain high levels of ROS, and when the NPs reach their target cells, the excessive ROS in the cells can cleave the boric acid ester–dextran bonds to release drugs specifically. Therefore, the PR-M2/BED@BA group presented the strongest pharmacological activity against oxidative stress.

### Anti-apoptotic effects

**Figure 8.**
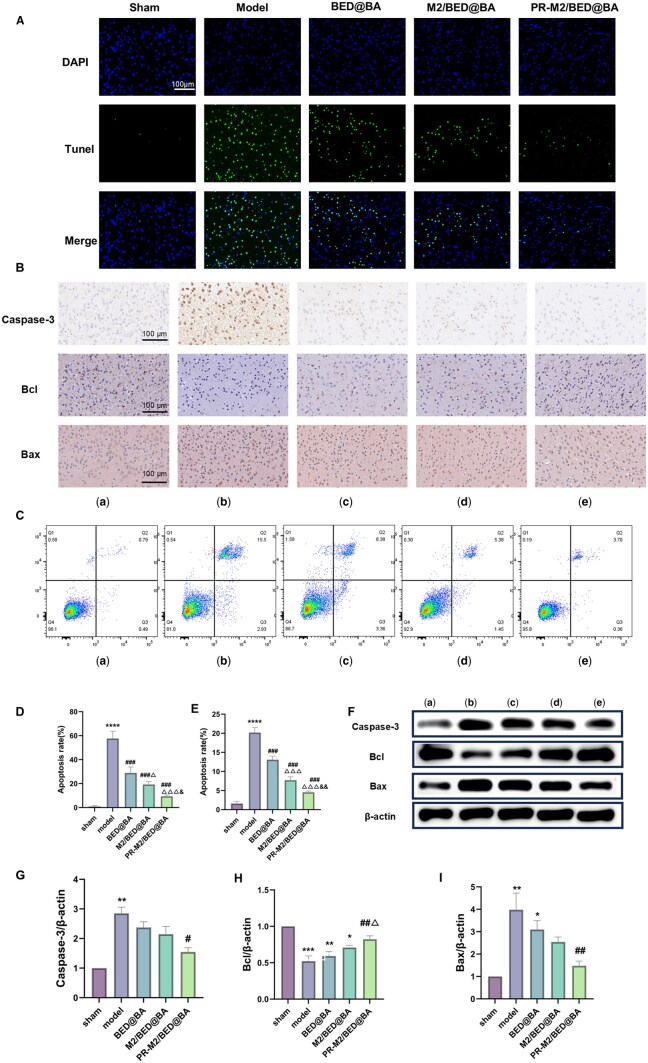
PR-M2/BED@BA attenuates neuronal apoptosis. (**A**) TUNEL staining of the brain of BCAL mice treated with different NPs (*n* = 4). (**B**) Immunohistochemistry of Caspase-3, Bcl and Bax protein (*n* = 4). (**C**) Apoptosis rate of PC12 cells detected by FCM (*n* = 4). (**D**) ImageJ software was used to quantify the proportion of apoptotic cells in (A) (*n* = 4). (**E**) Quantification of the percentage of apoptotic PC12 cells in (**C**) (*n* = 4). (**F**) WB detection of the expression of the apoptotic proteins Caspase-3, Bcl and Bax in the brain tissues of the BCAL mice. ImageJ software was used to semiquantitatively analyze the grayscale values of Caspase-3 (**G**), Bcl (**H**) and Bax (**I**) (*n* = 3). groups: (a) Sham (b) Model (c) BED@BA (d) M2/BED@BA (e) PR-M2/BED@BA. ****P < 0.0001, ***P < 0.001, **P < 0.01 and *P < 0.05 vs. sham group; ^####^*P* < 0.0001, ^##^*P* < 0.01 and ^#^*P* < 0.05 vs. model group; ^△△△^*P* < 0.001 and ^△^*P* < 0.05 vs. BED@BA group; ^&&^*P* < 0.01 and ^&^*P* < 0.05 vs. M2/BED@BA group.

Increasing evidence has shown that microglia are important factors contributing to the neuronal damage caused by CIR injury. Activated microglia release many inflammatory mediators and ROS, which in turn affect the survival and function of the surrounding neurons [43]. Regulating microglial M2 polarization and inhibiting microglial oxidative stress can reduce OGD/R-induced neuronal death [44, 45]. PR-M2/BED@BA promoted M2 microglial polarization to reduce inflammation and oxidative stress. Therefore, we further explored whether these NPs can exert neuroprotective effects by regulating microglia. As shown in Figure 8C and E, the percentage of apoptotic PC12 cells in the model group was 18.43%, which significantly differed from that in the sham group. Compared with the model group, BED@BA and M2/BED@BA significantly reduced the percentage of apoptotic cells by 11.74% and 6.83%, respectively, and PR-M2/BED@BA further significantly reduced the percentage of apoptotic cells to 4.06%. The TUNEL results (Figure 8A and D) revealed that, compared with the number in the sham group, the number of green fluorescent cells in the model group was significantly greater. After administration, the different NPs reduced the number of apoptotic cells to varying degrees, and PR-M2/BED@BA had the best antiapoptotic effect. When cells sense apoptotic signals, the ratio of the antiapoptotic protein Bcl2 to the proapoptotic protein Bax increases, resulting in the release of cytochrome c, which activates caspase-3, thereby initiating apoptosis. Therefore, we further detected the expression of apoptosis-related proteins by WB (Figure 8F). Compared with the model group, PR-M2/BED@BA significantly reduced the expression of the Caspase-3 and Bax proteins in the brain tissues of the BCAL mice and significantly increased Bcl protein expression. In addition, we performed immunohistochemistry to detect the expression of the apoptosis-related proteins Caspase-3, Bcl and Bax in the cerebral cortex of the mice, and the results were consistent with the WB results (Figure 8B). In summary, the experimental results show that PR-M2/BED@BA has the best neuroprotective effect, which is attributed mainly to its indirect protection of neuronal cells via the regulation of microglial polarization and oxidative stress.

### *In vivo* safety

**Figure 9.**
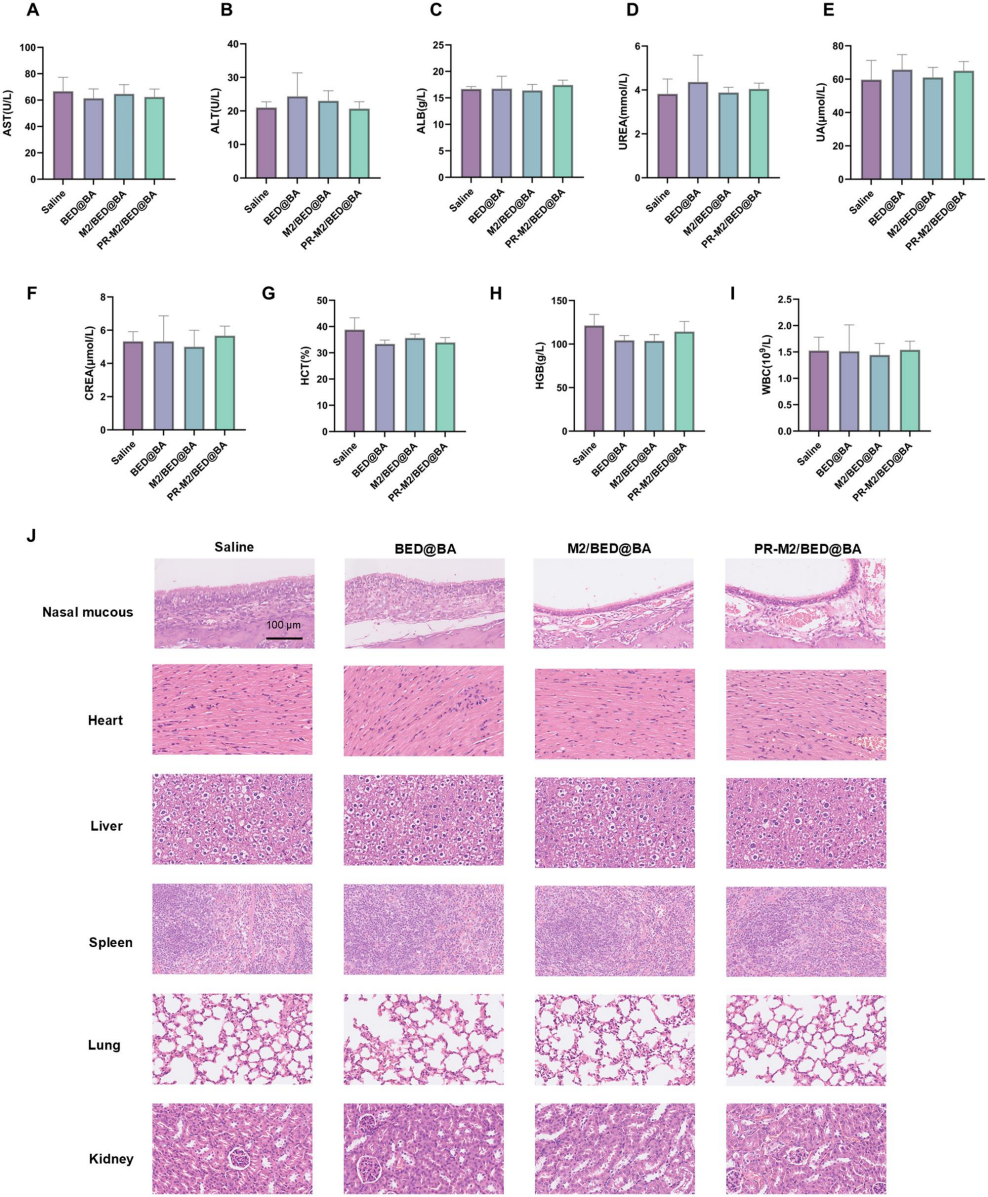
The *in vivo* safety of PR-M2/BED@BA. Liver function indexes AST (**A**), ALT (**B**), ALB (**C**), renal function indexes UREA (**D**), UA (**E**), CREA (**F**) and blood routine indexes HCT (**G**), HGB (**H**), WBC (**I**). (*n* = 3) (**J**) The histochemical analysis of the nasal mucosa, brain, heart, liver, spleen, lung and kidney tissue sections was performed by HE staining (*n* = 3).

As a method to verify the safety of the NPs delivered to the brain after nasal administration, HE staining was performed to observe the pathological changes in the nasal mucosa and tissues of each group of mice. Figure 9J shows that the tissues from each NP group were similar to those from the saline group. The nasal mucosa tissue structure of the mice in each group was intact, the mucosal surface was covered with normal pseudostratified columnar cilia, and the mucosal epithelial cells were closely arranged. The tissue morphology and structure of the heart, liver, spleen, lung and kidney of the mice in each group also remained essentially unchanged, and the structures of the cells and nuclei were intact. Overall, no obvious abnormal pathomorphological changes were found. These findings indicated that BED@BA, M2/BED@BA and PR-M2/BED@BA had no significant effects on the nasal mucosa, heart, liver, spleen, lungs or kidneys of normal mice after continuous administration at therapeutic doses for 7 days. In addition, we monitored the blood parameters and blood biochemistry of each NP-treated group. As expected, the hematological parameters of the BED@BA, M2/BED@BA and PR-M2/BED@BA groups did not differ significantly from those of the saline group (Figure 9A–I). These results reveal the therapeutic biosafety of PR-M2/BED@BA.

## Conclusion

In summary, we constructed a multitargeted nasal-targeted nanodrug delivery system (PR-M2/BED@BA) for the efficient treatment of CIR injury. After nasal administration, the surface-camouflaged PR-modified M2 microglial membranes allowed PR-M2/BED@BA to cross the nasal mucosal barrier and home to ischemic areas, actively targeting the brain lesion site and accumulating in microglia and neuronal cells. The NP core shows ROS-responsive characteristics, scavenges excessive ROS in the pathological environment and responds by releasing BA to exert neuroprotective effects. In both *in vitro* and *in vivo* experiments, PR-M2/BED@BA significantly improved the nasal–brain delivery efficiency, microglia-targeting ability and neurobehavioral scores after alleviating the CIR-induced pathological damage to the brain tissue. These neuroprotective effects are related to the regulation of microglial M1-type to M2-type polarization to reduce inflammation, decrease microglial oxidative stress, and thus, lower neuronal apoptosis. Therefore, this nanocarrier provides new ideas for the design of nasal–brain drug delivery systems and a safe and effective treatment strategy for neurological diseases such as CIR injury.

## Supplementary Material

rbaf063_Supplementary_Data
