# Supplementary material for: Functionalized biomimetic nanoparticles are delivered from the nose to the brain for the synergistic targeted treatment of cerebral ischemia/reperfusion injury
Source: Regen Biomater. 2025 Jun 27;12:rbaf063. doi: 10.1093/rb/rbaf063 (PMC12459241; doi:10.1093/rb/rbaf063)
Supplement: rbaf063_Supplementary_Data [file rbaf063_supplementary_data.zip › Supplementary data.docx]

**Accessory materials**

**Characterization by 1H NMR and FTIR**

1. ^1^H NMR

The structure of BED was characterized by ^1^H-NMR at 600 mhz using Bruker Avance spectrometer with deuterated methanol and deuterated DMSO as solvents at a volume ratio of 2:1, and the results were analyzed by MestReNova software.

1. FTIR

BA, PR, M2-BV2 cell membrane, BED@BA, M2/BED@BA, PR-M2/BED@BA, dextran and BED were prepared by KBr disk method and analyzed by Fourier transform infrared spectrophotometer. The sample is scanned in the range of 4000 to 400 wavenumbers (cm^-1^).

**CCK8**

Different concentrations of PR-M2/BED@BA (10, 50, 100, 150, 200 μg/ml) were added to BV2, PC12 and Calu-3 cells. After 24 hours of incubation, CCK8 was used to detect cell viability.

**Determination of encapsulation efficiency and drug loading**

PR-M2/BED@BA was prepared according to the above method. After demulsification with formamide and methanol, the encapsulation efficiency and drug loading of PR-M2/BED@BA were detected by ultraviolet spectrophotometer (UV-6100S, Mapata, China). Its calculation formula is as follows:

Drug loading% = (total mass of loaded nano-drug/total mass of co-loaded nano-drug) ×100%

Encapsulation efficiency% = (total mass of loaded nano-drug / total mass of theoretical input drug) ×100%

***P*_app_ calculation**

$$\boldsymbol{P}_{\boldsymbol{app}}\boldsymbol{=}\frac{\boldsymbol{ⅆQ/ⅆt}}{\boldsymbol{AC}}$$

In the formula, *P*_app_ is the apparent permeability coefficient (cm/s); *dQ*/*dt* is the drug transport rate; A is the area of polycarbonate membrane (1.12 cm^2^); C is the initial concentration of coumarin 6 in the upper chamber of transwell (20 μg/mL).

**BED scavenges ROS in vitro**

BV2 cells were subjected to OGD/R modeling. During reoxygenation, BA, BED@BA (equivalent to 5 μg/mL BA) and BED (200 μg/mL) were added to the cell culture medium and incubated with BV2 cells for 24 h. The cell culture medium was removed, and the appropriately diluted fluorescent probe DCFH-DA (1:1000) was added. The cells were incubated in a cell culture box at 37°C for 30 minutes, and the cells were washed three times with PBS. BV2 cells were collected and intracellular ROS was detected by flow cytometry.

1. **IL-4 induced M2 microglial cells**


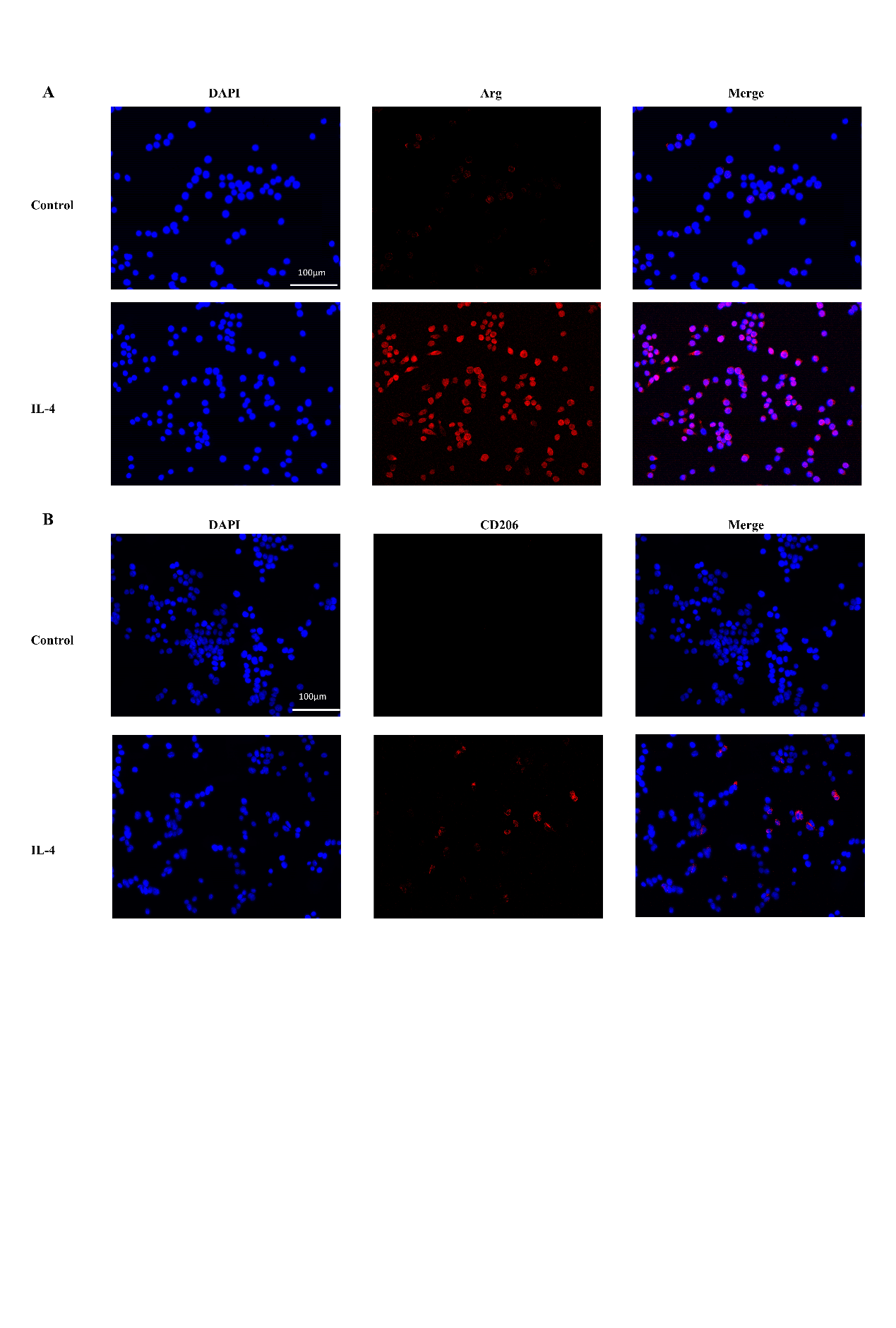


Figure S1. (A) Immunofluorescence was used to detect the expression of Arg in BV2 cells induced by IL-4. (B) Immunofluorescence was used to detect the expression of CD206 in BV2 cells induced by IL-4 (n=4).

1. **The synthesis steps of BED**


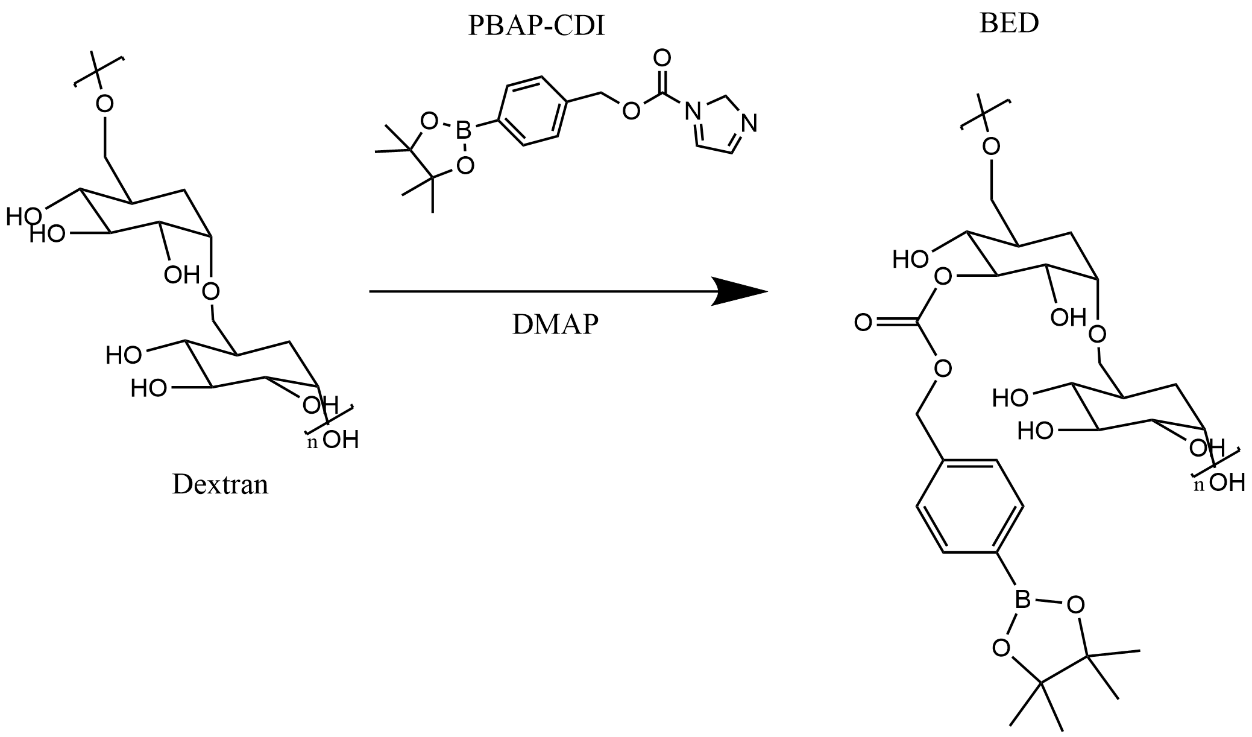


Figure S2. The synthesis steps of BED.

1. ^
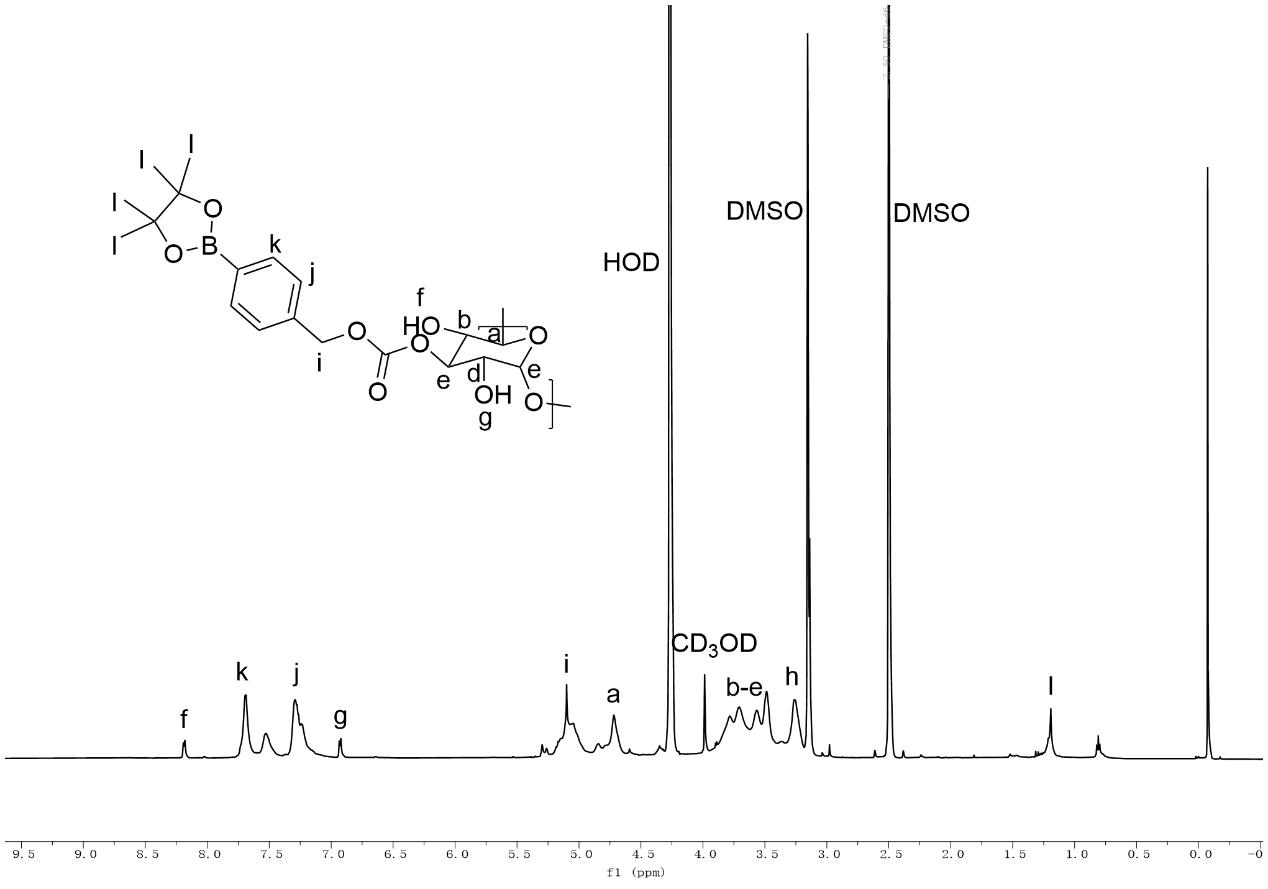
^**^1^****H-NMR**

Figure S3. ^1^H-NMR of BED

1. **FTIR**


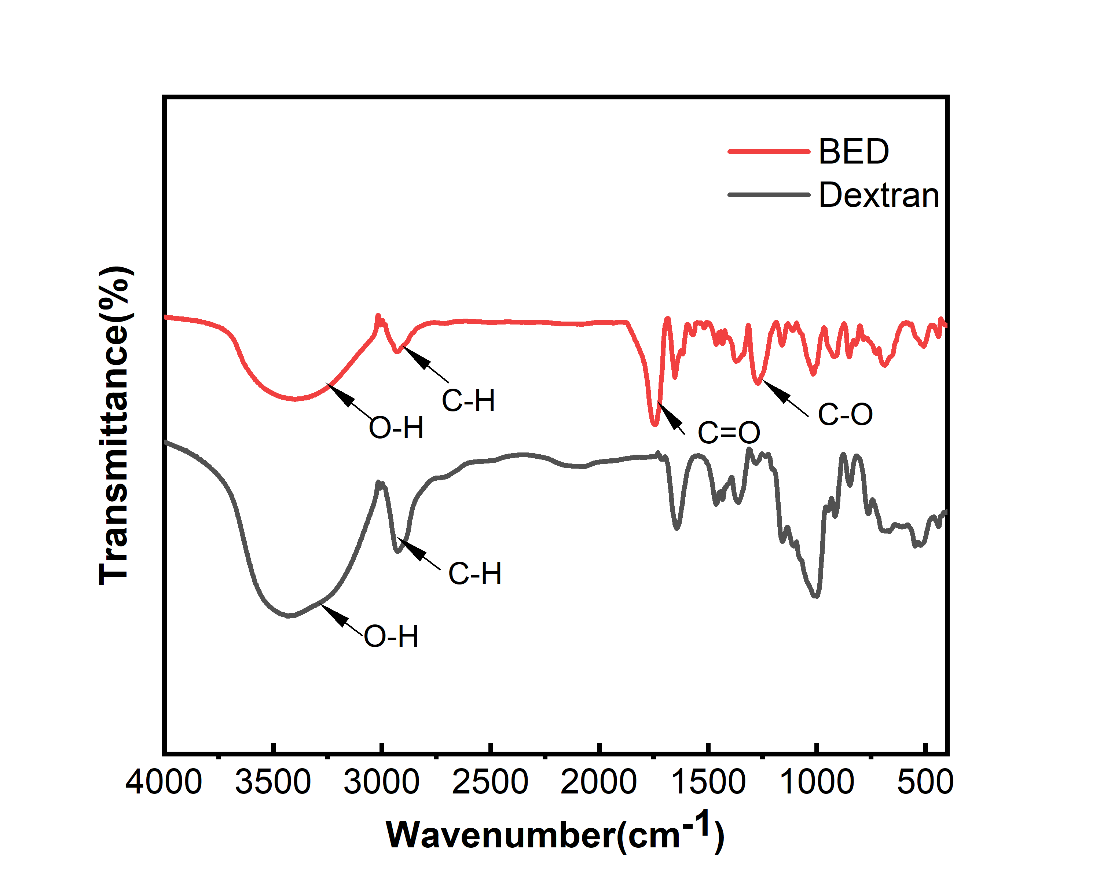


Figure S4. FTIR of BED and dextran

1. **FTIR**


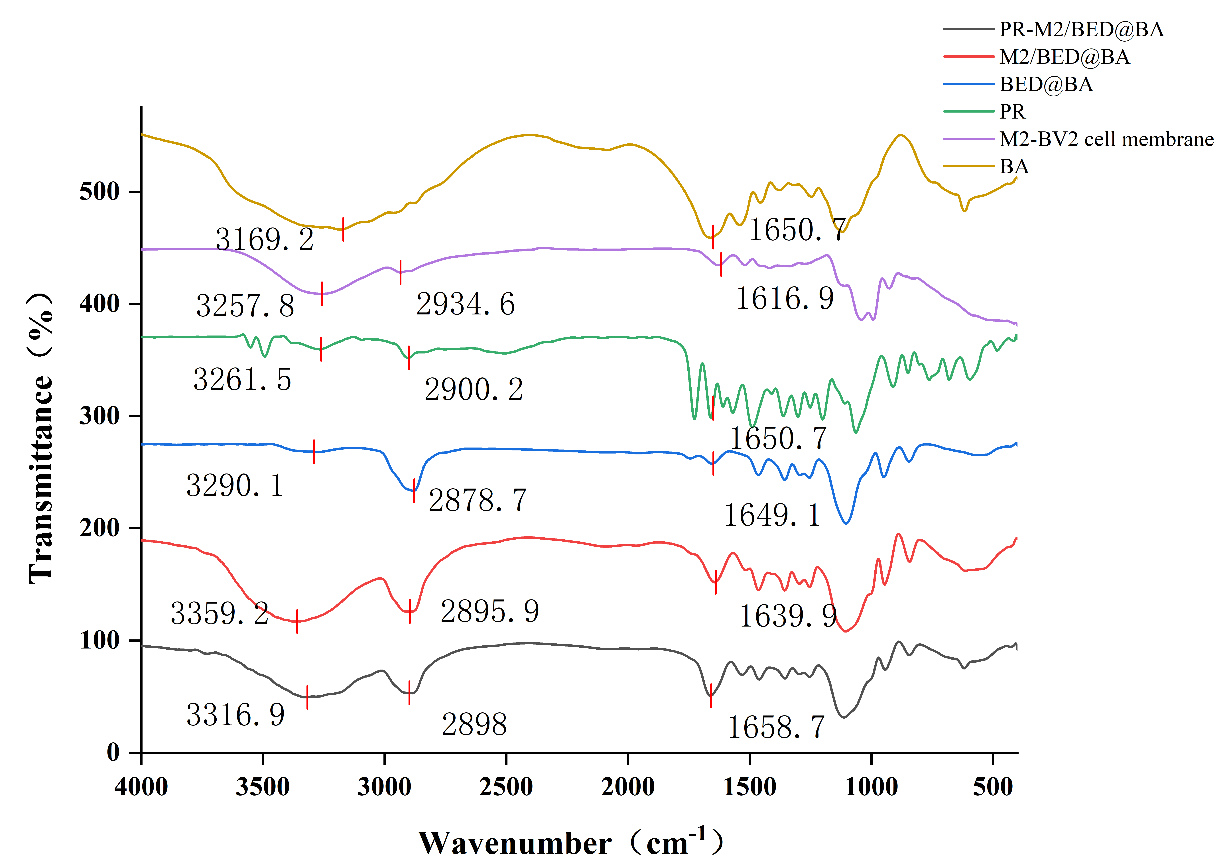


Figure S5. FTIR of NPs.

1. **Determination of encapsulation efficiency and drug loading**

Tabble S1. Encapsulation efficiency and drug loading determination results

| Sample | Encapsulation efficiency（%） | Drug loading  （%） |
| --- | --- | --- |
| BED@BA | 56.7% | 2.58% |
| M2/BED@BA | 57.1% | 2.64% |
| PR-M2/BED@BA | 62.9% | 2.51% |

1. **CCK8**


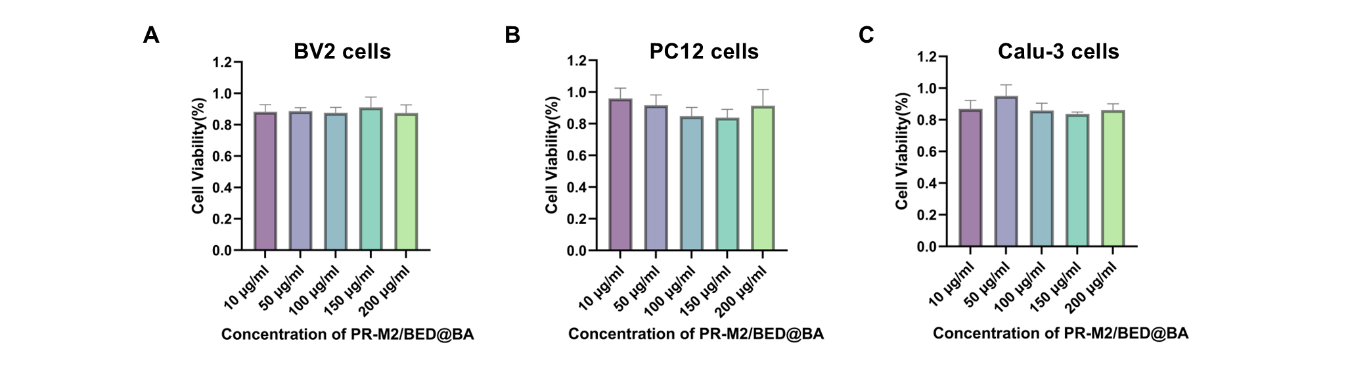


Figure S6. The effect of different concentrations of PR-M2/BED@BA on the viability of BV2 cells(A), PC12 cells(B) and Calu-3 cells(C). (n=4)

1. **ROS responsiveness**


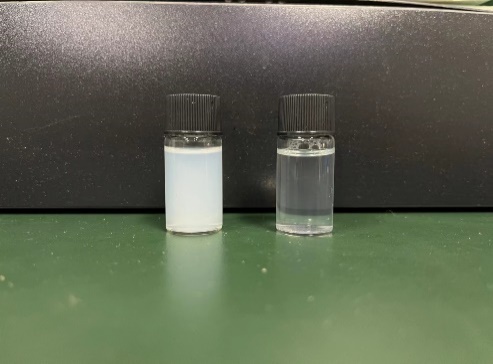

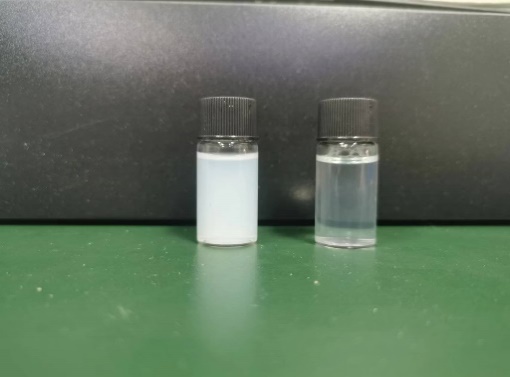

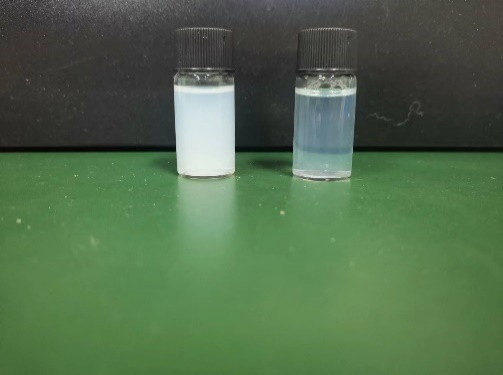

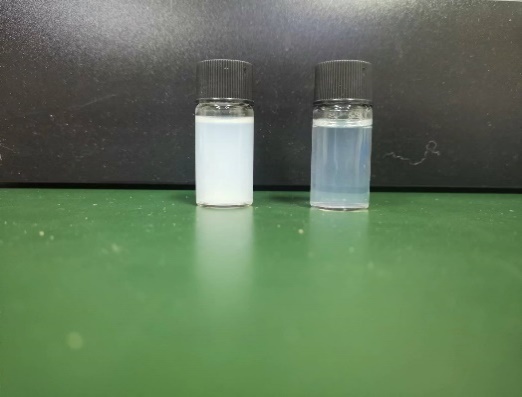

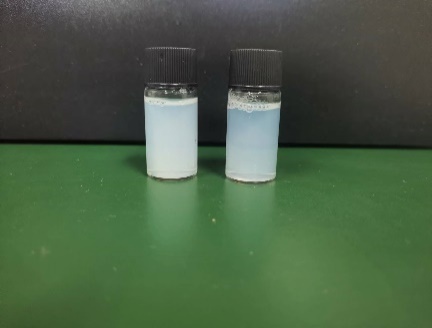

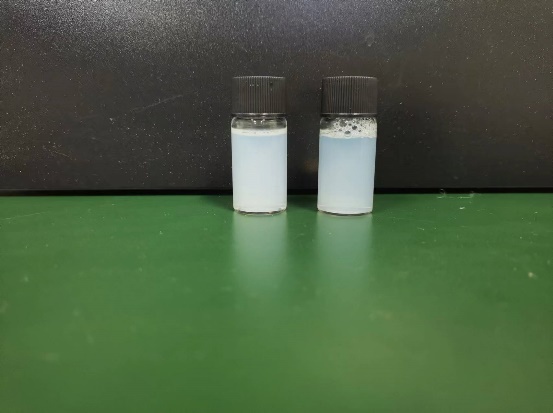


0 min 5 min 15 min 30 min 1 h 2 h

Figure S7. Time-dependent degradation of PR-M2/BED@BA in PBS with H_2_O_2_ (1 mM).


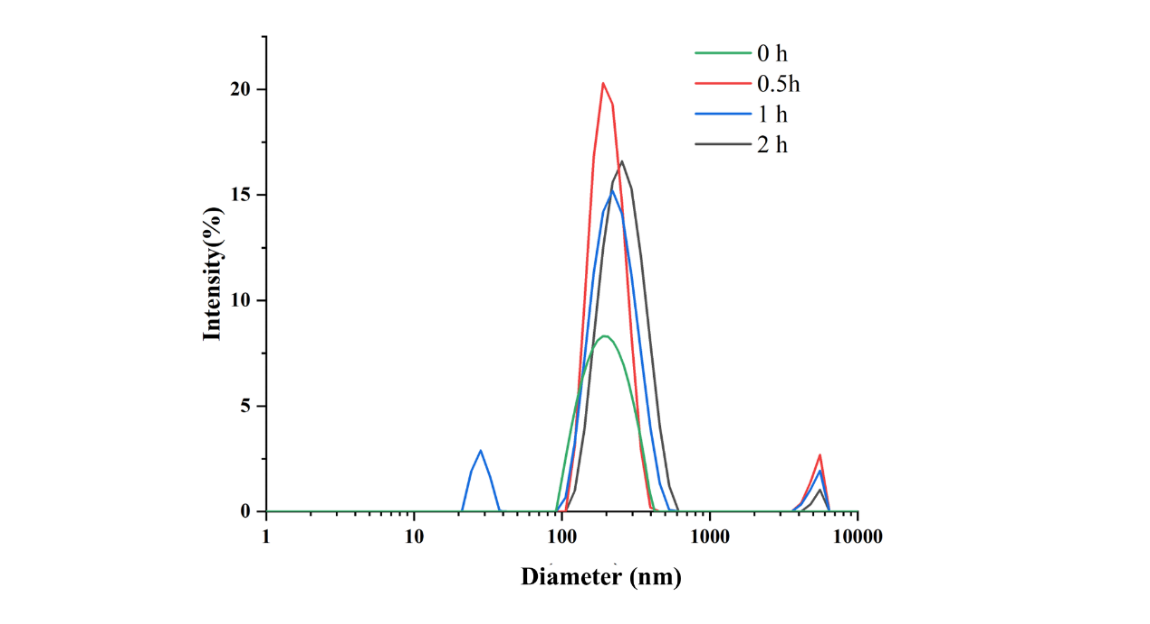


Figure S8. The diameters change of PR-M2/BED@BA in H_2_O_2_ (1 mM) at different time points.

Tabble S2. The change of diameter and PDI of PR-M2/BED@BA after incubation in H_2_O_2_ at different time points.

| Time (h) | Diameter（nm） | PDI |
| --- | --- | --- |
| 0 | 191.2±4.98 | 0.186±0.004 |
| 0.5 | 238.1±5.88 | 0.25±0.163 |
| 1 | 326.3±8.33 | 0.32±0.008 |
| 2 | 424.4±15.6 | 0.36±0.016 |


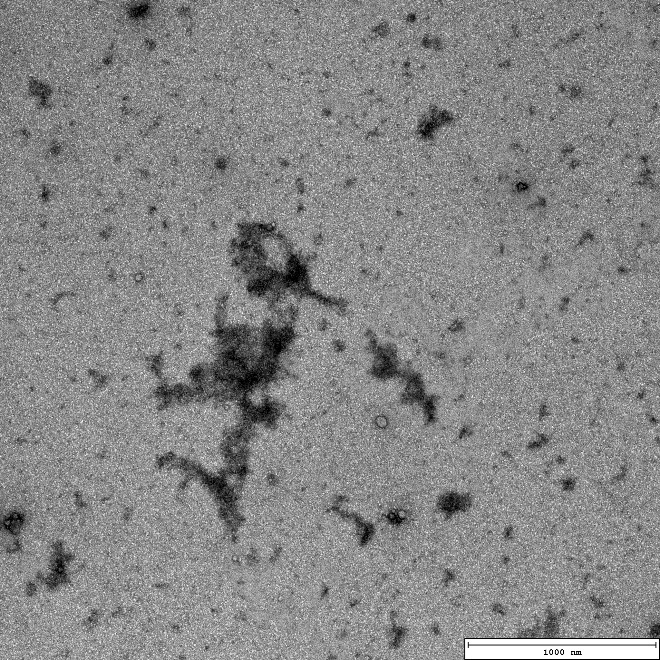
Figure S9.TEM image of PR-M2/BED@BA after incubation with H_2_O_2_ (1 mM) for 2 h.

1. **The stability of PR-M2/BED@BA**


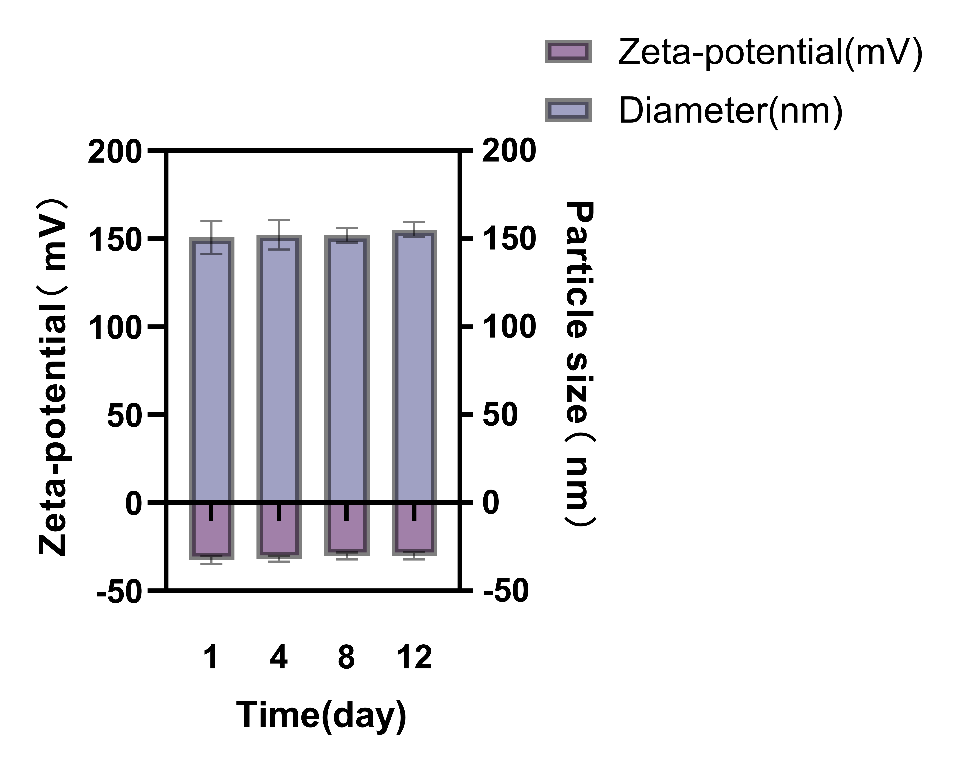


Figure S10. The diameters and zeta-potential of PR-M2 / BED @ BA within 12 days. (n=3)

1. **Scavenging of ROS**


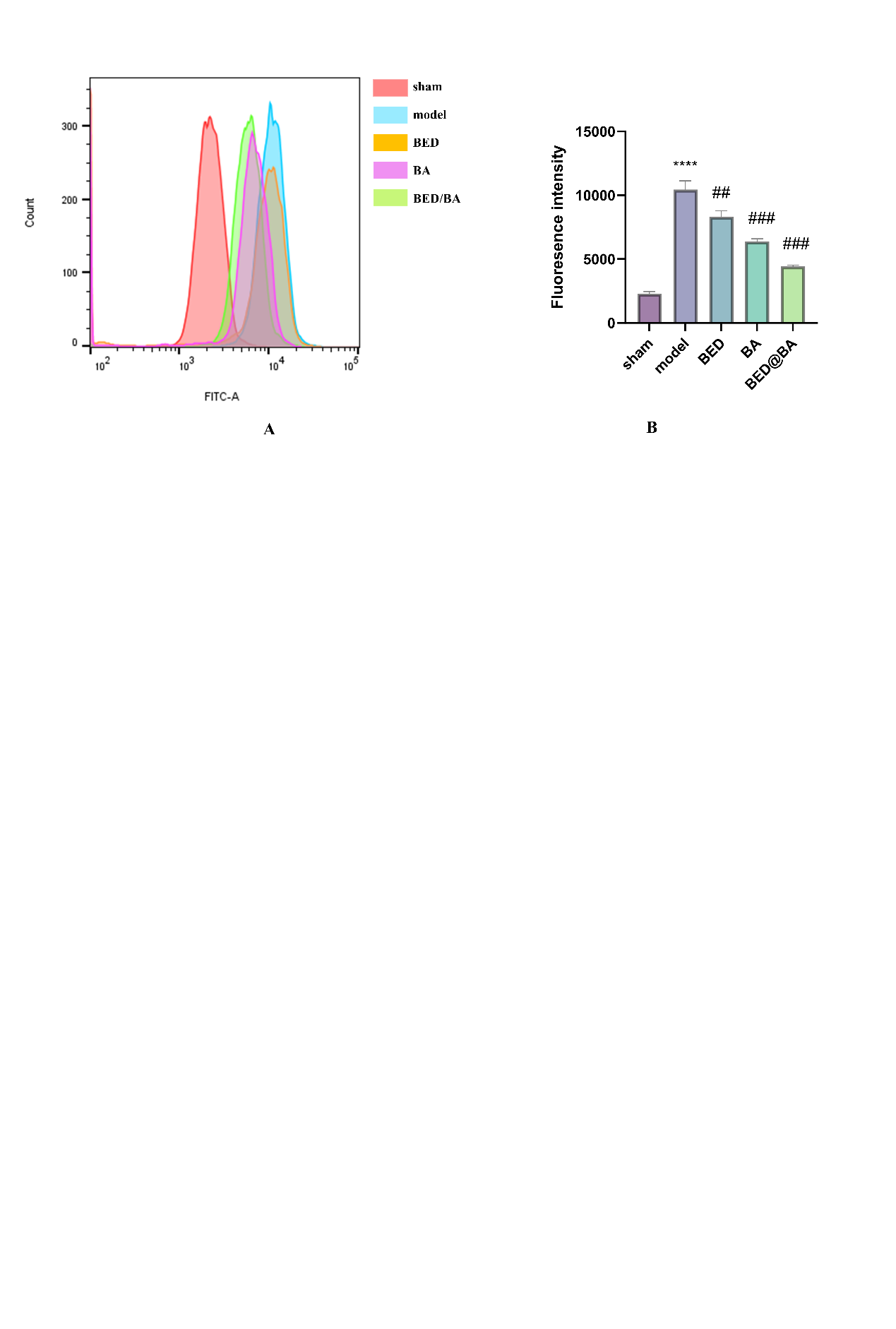


Figure S11. (A)Flow cytometry analysis of ROS after OGD/R in BV2 cells. (B) Quantitative analysis of (A). (n=4)
